# Facility-Based Delivery during the Ebola Virus Disease Epidemic in Rural Liberia: Analysis from a Cross-Sectional, Population-Based Household Survey

**DOI:** 10.1371/journal.pmed.1002096

**Published:** 2016-08-02

**Authors:** John Ly, Vidiya Sathananthan, Thomas Griffiths, Zahir Kanjee, Avi Kenny, Nicholas Gordon, Gaurab Basu, Dale Battistoli, Lorenzo Dorr, Breeanna Lorenzen, Dana R. Thomson, Ami Waters, Uriah G. Moore, Ruth Roberts, Wilmot L. Smith, Mark J. Siedner, John D. Kraemer

**Affiliations:** 1 Medical Team, Last Mile Health, Zwedru, Liberia; 2 Monitoring and Evaluation Team, Last Mile Health, Zwedru, Liberia; 3 Division of Global Health Equity, Brigham and Women’s Hospital, Boston, Massachusetts, United States of America; 4 Cambridge Health Alliance, Cambridge, Massachusetts, United States of America; 5 Harvard Medical School, Boston, Massachusetts, United States of America; 6 Implementation Team, Last Mile Health, Cestos City, Liberia; 7 Department of Global Health and Social Medicine, Harvard Medical School, Boston, Massachusetts, United States of America; 8 Rivercess County Health Team, Liberian Ministry of Health and Social Welfare, Cestos City, Liberia; 9 Center for Global Health, Massachusetts General Hospital, Boston, Massachusetts, United States of America; 10 Department of Health Systems Administration, Georgetown University, Washington, DC, United States of America; 11 African Studies Program, Georgetown University, Washington, DC, United States of America; Harvard University, UNITED STATES

## Abstract

**Background:**

The Ebola virus disease (EVD) epidemic has threatened access to basic health services through facility closures, resource diversion, and decreased demand due to community fear and distrust. While modeling studies have attempted to estimate the impact of these disruptions, no studies have yet utilized population-based survey data.

**Methods and Findings:**

We conducted a two-stage, cluster-sample household survey in Rivercess County, Liberia, in March–April 2015, which included a maternal and reproductive health module. We constructed a retrospective cohort of births beginning 4 y before the first day of survey administration (beginning March 24, 2011). We then fit logistic regression models to estimate associations between our primary outcome, facility-based delivery (FBD), and time period, defined as the pre-EVD period (March 24, 2011–June 14, 2014) or EVD period (June 15, 2014–April 13, 2015). We fit both univariable and multivariable models, adjusted for known predictors of facility delivery, accounting for clustering using linearized standard errors. To strengthen causal inference, we also conducted stratified analyses to assess changes in FBD by whether respondents believed that health facility attendance was an EVD risk factor. A total of 1,298 women from 941 households completed the survey. Median age at the time of survey was 29 y, and over 80% had a primary education or less. There were 686 births reported in the pre-EVD period and 212 in the EVD period. The unadjusted odds ratio of facility-based delivery in the EVD period was 0.66 (95% confidence interval [CI] 0.48–0.90, *p*-value = 0.010). Adjustment for potential confounders did not change the observed association, either in the principal model (adjusted odds ratio [AOR] = 0.70, 95%CI 0.50–0.98, *p* = 0.037) or a fully adjusted model (AOR = 0.69, 95%CI 0.50–0.97, *p* = 0.033). The association was robust in sensitivity analyses. The reduction in FBD during the EVD period was observed among those reporting a belief that health facilities are or may be a source of Ebola transmission (AOR = 0.59, 95%CI 0.36–0.97, *p* = 0.038), but not those without such a belief (AOR = 0.90, 95%CI 0.59–1.37, *p* = 0.612). Limitations include the possibility of FBD secular trends coincident with the EVD period, recall errors, and social desirability bias.

**Conclusions:**

We detected a 30% decreased odds of FBD after the start of EVD in a rural Liberian county with relatively few cases. Because health facilities never closed in Rivercess County, this estimate may under-approximate the effect seen in the most heavily affected areas. These are the first population-based survey data to show collateral disruptions to facility-based delivery caused by the West African EVD epidemic, and they reinforce the need to consider the full spectrum of implications caused by public health emergencies.

## Abstract 2

John Kraemer and colleagues estimate changes in facility-based delivery during the Ebola virus disease epidemic in rural Liberia using cross-sectional data from a population-based household survey.

## Author Summary

**Why was this study done?:**

- The 2014–2015 West African Ebola virus disease (EVD) epidemic killed approximately 11,300 people directly, but its effects are magnified by collateral health consequences.
- Mathematical models and facility-based data provide evidence of substantial health service interruptions, but little population-based survey data has been published from the three principally affected countries.
- Maternal health services, which are dependent on functioning health systems, are likely to be particularly susceptible to external shocks, such as the EVD epidemic.

**What did the researchers do and find?:**

- We used a cluster-sample survey to produce a representative sample of births in Rivercess County, Liberia, a part of the country with relatively limited Ebola transmission.
- Controlling for potential confounders, we compared the odds of facility-based delivery among 686 births in the period before the EVD epidemic with 212 births during the epidemic.
- We identified a 30% reduction in the odds of facility-based delivery during the EVD epidemic. The odds of facility-based delivery were 41% lower among women who reported a belief that Ebola was or may be transmitted in health facilities, but not significantly lower among women who reported believing that Ebola was not transmitted in health facilities.

**What do these findings mean?:**

- This study provides further evidence that the 2014–2015 West African EVD epidemic caused serious collateral harm to health services.
- This study also underscores the need to maintain focus on long-term health system reconstruction after the EVD epidemic.

## Introduction

The 2014–2015 Ebola virus disease (EVD) epidemic killed approximately 11,300 people in West Africa, with sporadic cases continuing to be reported [1]. While these numbers dwarf all prior hemorrhagic fever epidemics, EVD-related morbidity and mortality have been hypothesized to represent only a small fraction of the epidemic’s overall effect on health in the region. Long-term consequences of the epidemic will likely be exacerbated by the loss of over 500 healthcare workers who have died from EVD in the three most affected countries, all of which suffered from pre-existing healthcare worker shortages [2]. In the shorter-term, innumerable health facilities closed or interrupted access to preventive and therapeutic services for non-Ebola conditions [3–6]. Perhaps as disruptive to healthcare access was widely documented EVD-related stigma and fear of EVD transmission within health facilities, both of which have been hypothesized to decrease demand for services [7–9]

Recent work has sought to measure these indirect effects of the epidemic on health and health care access. [7,10–22] Both modeling studies and analyses of health facility data have begun to shed light on the epidemic’s indirect health consequences, but are limited by assumptions and difficulties disentangling the epidemic’s effects on service utilization and routine data collection. A more precise examination of the collateral consequences to health caused by the Ebola epidemic will serve multiple purposes, including (1) identifying vulnerable points within health systems in resource-limited settings during public health emergencies, (2) improving our understanding of the scope of such shocks, and, perhaps most importantly, (3) signaling to the global community the need for persistent dedication to health systems rebuilding even after the last cases have been detected [23–26]

Because effective maternal healthcare delivery is dependent on functioning health facilities and trained personnel, it represents an area of public health particularly susceptible to shocks. Prior to the Ebola epidemic, Liberia had made improving maternal health a national priority, which resulted in substantial gains in facility-based delivery (FBD) rates. Nationally, FBD rates increased from 38% to 56% between the 2007 and 2013 demographic and health surveys (DHS). The proportionate increase was even greater in rural areas, where rates improved from 26% to 46% [27,28]. In sub-Saharan Africa, FBD is a key component of the maternal health service cascade, associated with substantial improvements in maternal and neonatal outcomes when appropriate clinical functions are provided at adequate quality [29–33]. Additionally, prior birth location tends to predict delivery location for future pregnancies when services are adequate [34,35,] and FBD is an important step in the continuum of care to post-natal and subsequent young child services [36]. As such, if the Ebola epidemic has reduced FBD, long-term ripple effects would be expected for affected mothers and children.

We analyzed data from a community-based household survey conducted in Rivercess County, Liberia, during March and April, 2015. The survey was designed as a baseline assessment prior to implementation of a community health worker (CHW) program. Survey questions included a complete birth history and location for all deliveries. We constructed a retrospective cohort of deliveries from the cross-sectional survey to estimate FBD rates before and during the Ebola epidemic. The primary aim of this study was to estimate the effect of Ebola on healthcare utilization by assessing changes in FBD in a rural Liberian setting before and during the 2014–2015 epidemic.

## Methods

### Ethics

This study was approved by the ethics review boards at the Liberian Institute for Biomedical Research, Georgetown University, and Partners Healthcare. All participants provided verbal informed consent prior to participation.

### Setting, Sampling Approach, and Survey Administration

Rivercess County is a rural county located in south-central Liberia with approximately 71,500 residents as of the 2008 national census [37]. It had limited Ebola transmission, with 34 confirmed or probable cases reported to the World Health Organization, principally linked to a single cluster in October–November 2014 [38–41]. Participants were sampled using a stratified, two-stage cluster-sample approach. The sample was stratified for purposes of the survey’s function as baseline for a stepped-wedge impact evaluation, with two strata corresponding to the intervention’s phased implementation. The third stratum included areas within 5 kilometers (km) of a health facility, where CHWs are not deployed by current Liberian national policy, and which were assessed to provide county-wide estimates for health officials.

Prior to sampling, we enumerated all households in each village in the county. At the first stage, we sampled villages with probability proportionate to size within each stratum using the standard DHS approach: listing clusters with a running cumulative number of households, determining the sampling interval necessary to take the correct number of clusters, randomly determining a starting value, and then selecting each subsequent cluster that corresponded to the sampling interval [42–44]. At the second stage, 21 households were selected per cluster in compact segments by (1) spinning a laminated paper triangle on the ground in the village’s center, (2) using a random number generator to randomly select an initial house in the direction pointed between the center and margin of the village, and (3) continuing to the next closest dwelling until 21 households were sampled [45,46]. We surveyed all women aged 18 to 49 in each selected household.

The survey (see S1 Survey) included questions on household wealth, including asset ownership, water and toilet facilities, and housing materials, and maternal health, drawn principally from the 2013 Liberian Demographic and Health Survey [27]. A section on Ebola knowledge, attitudes, and practices was produced by the research team. All questions were translated from American English to Liberian vernacular English and back-translated by bilingual staff to ensure accuracy. Because some respondents were expected to speak only Bassa, a local language without a commonly used written form, bilingual enumerators administered the survey. Prior to survey administration, all enumerators attended a five-day training, which included practice administering the survey as well as training on informed consent, proper use of the mobile platform, survey skip logic, and techniques to reduce bias [47]. Field supervisors (with one supervisor per three-enumerator field teams) observed implementation of surveys daily and ensured quality assurance at the point of survey implementation, and one additional supervisor oversaw all field teams to ensure consistency. Data were entered using Commcare, an Android-based mobile platform, and maintained in a MySQL database, with basic data cleaning conducted prior to exportation for analysis.

### Measures

Our primary outcome of interest, having a facility-based delivery, was recorded for all respondents with at least one prior pregnancy. Our primary predictor of interest, whether the delivery occurred during the EVD period, was generated from child dates of birth. June 15, 2014, was chosen to dichotomize the periods before and during the epidemic, because it was the approximate date on which Ebola re-emerged as a broadly perceived national threat and when the first media reports emerged that patients were avoiding health facilities [48–54], and because, in limited published hospital record data from elsewhere in the country, June is the earliest month in which there appears a reduction in facility visits [15,16]. The pre-EVD comparison period began 4 y prior to the beginning of survey administration.

In our models, we included potential confounding variables that have been identified as determinants of FBD in prior studies [30,34,45]. These included whether the birth occurred in the rainy or dry season (rainy season is May to October [55]), maternal marital status, household language (Liberian English versus Bassa), birth order (categorized as first, second or third, and fourth or higher), and self-reported maternal education (categorized as none, primary school only, and any secondary school or higher). Maternal age at each birth was calculated by measuring elapsed time between date of birth and the mother’s reported age when surveyed. Because prior studies [30,34,45] have found no consistent relationship between maternal age and FBD to give us an a priori basis on how to include maternal age, we categorized it into quartiles. A household wealth index was constructed using the standard DHS approach of assessing household assets, housing quality, water source, and toilet facilities [56]. The index was constructed using principal components analysis and then assigning relative percentiles of wealth. Prior work has demonstrated that FBD increases consistently with greater wealth [27,28,57]; we investigated the association between FBD and wealth in the pre-Ebola period using locally weighted scatterplot smoothing (LOWESS), which confirmed that the relationship between wealth percentiles and FBD was logit-linear (see S1 Fig). As a result, we included wealth as a continuous variable, rescaling it by dividing by its own interquartile range for ease of interpretation. Road distance from the center of each cluster to the nearest health facility was measured by global positioning systems (GPS) devices (Garmin eTrex 10; Garmin Ltd.). Prior research has shown complex inverse relationships between distance and FBD [45,58–61], so we examined the relationship using LOWESS plots, which suggested distance appeared to have a logit-linear, splined relationship with nodes at 10 and 21 km (see supplemental S1 Fig).

Finally, to assess the causality of a relationship between time period and FBD, we included a survey item about whether respondents believed health facilities to be a source of Ebola transmission. We dichotomized this variable by combining respondents who stated they believed health facilities posed a definite or uncertain risk versus those who stated facilities posed no Ebola risk.

### Statistical Methods

We used standard summary statistical methods to describe respondents’ demographic and socioeconomic characteristics. We tested differences in respondents’ characteristics before versus during the Ebola period using design-corrected chi-squared analysis for categorical variables. Differences in the distribution of continuous variables, which were not normally distributed, were tested using Somers’ D, an analogue to the Mann-Whitney *U* test that can accommodate complex sample survey data [62,63].

We fit design-corrected logistic regression models to estimate associations between giving birth during the Ebola epidemic and FBD. While we had a priori bases to expect certain variables to be associated with FBD, we lacked a theoretical basis to expect particular variables to be associated with the EVD period and, therefore, to constitute potential confounders [64]. Therefore, we examined bivariate associations and constructed three multivariable models to assess for associations between the pre- and intra-EVD time period and FBD. The bivariate model assumes that Ebola is completely exogenous, so it presents only unadjusted relationships. In multivariable model 1, which serves as our primary model, we included only those predictors that were associated with both the EVD period and FBD during the pre-Ebola period at or below the *p* = 0.10 level [57]. Multivariable model 2 includes all variables associated with either Ebola or FBD at or below the *p* = 0.10 level. Multivariable model 3 includes all considered variables identified in the literature as potentially associated with FBD. Multivariable models 2 and 3 principally serve as robustness checks.

We plotted levels of FBD in the post-Ebola period compared to preceding years adjusted for the controls in multivariable model 1 using predictive margins with covariates held at their observed levels. As a robustness check, we also graphically depicted FBD as a function of continuous calendar time using local polynomial regression (see S1 Appendix).

To test whether observed associations between time period and FBD might be due to secular trends unrelated to Ebola, we conducted a stratified analysis that investigated FBD rates among (1) those who reported a belief that health facilities were a definite or uncertain risk for Ebola transmission and (2) those who believed that health facilities were not a risk for Ebola transmission. Because health facilities did not close in Rivercess County during the epidemic, we considered fear to be the greatest Ebola-related barrier to healthcare utilization in this area [65]. As such, we hypothesized that if Ebola were causally related to alterations in FBD, we would expect to identify a lower rate of FBD during the EVD period among those who perceived that health facilities posed a risk for transmission, and a lesser reduction among those who did not hold this belief. We assessed for differences in FBD rates in these stratified sub-analyses using survey-design-corrected logistic regression.

All analyses accounted for our survey’s stratified design and incorporated clustering at the village and household levels. Taylor linearization was used to adjust standard errors for clustering in all parametric analyses; jackknifed errors were used with Somers’ D because it is not amenable to linearized errors. Because sampling probabilities differed by stratum, sampling weights were incorporated as the inverse probability of selection and corrected for non-response at the stratum level [42,66]. We incorporated finite population corrections at both sampling levels. Based on estimates of FBD rates and survey design effects from prior household-based surveys in rural Liberia [27,45], we estimated that approximately 870 births would be required to detect a 10% reduction in FBD with 80% power, which equated to approximately 4 y of birth data. We used Stata version 14 for all analyses. Replication datasets (S1 Dataset) and statistical code (S1 Code) are provided as online supplements. This study is reported per STROBE guidelines (S1 Checklist). Details of the analysis and any changes to the analysis plan are included in S1 Text.

### Sensitivity Analyses

We conducted several sensitivity analyses to assess for robustness to potential biases. First, because we did not survey currently hospitalized women, the survey risked underestimating FBD among recent births. We addressed this by fitting additional models that excluded women who gave birth within 2 wk before survey administration began. Second, we may have excluded births in the pre-EVD period to women who turned 50 before our survey was administered and therefore did not meet inclusion criteria. We addressed this with models restricted to births to women aged 45 or less so that the entire cohort met survey inclusion criteria. Third, we fit models excluding births from women who had moved since their last birth to avoid misallocation of household demographic variables. Fourth, we ran an analysis that combined the preceding three sensitivity analyses. Fifth, because the exact date when the perceived threat of EVD became salient is unknown (and likely varied between people), we varied our a priori definition of the start of the EVD epidemic. The three alternate dates chosen were: May 29, 2014, when Liberia’s second wave began; July 15, 2014, by which point Ebola transmission was widespread in the country; and August 6, 2014, when Liberia declared a national emergency. Sixth, because inclusion of more years in the control period may increase susceptibility to bias from secular trends, we ran analyses restricted to only 1 and 2 y of control data. Seventh, because date-of-birth heaping was observed for the first day of each month, we randomly redistributed these birth dates across each month, which was expected to have a mild effect on both inclusion and allocation between the pre-EVD and EVD periods. Finally, because variance estimation is sometimes sensitive to the approach chosen [67,68], we present findings with standard errors calculated by the jackknife method instead of linearization.

## Results

Ninety-four percent of intended households were surveyed. A total of 1,298 women out of 1,319 eligible respondents (98%) from 941 households completed a birth history. Missing data were rare: <1% of observations for all variables except belief about health facility transmission risk (5%). Median age at the time of survey was 29 y, and 86.3% had less than secondary education. Among the 898 births during the study period, 686 occurred during the pre-Ebola period and 212 in the Ebola period. Respondent characteristics were similar between the two periods (Table 1), except that the median wealth index score was lower among households with births in the Ebola period (*p* < 0.001), and primary school attendance was higher (*p* = 0.002).

**Table 1:** Demographic and social characteristics, Rivercess, Liberia, by births that occurred before and during the Ebola period.

	Pre-Ebola Period (March 2011–June 14, 2014)	Ebola Period Births (June 15, 2014–April 2015)	*p*-value
	*n* = 686	*n* = 212	
Household wealth index*, median (IQR)	-0.20 (-0.59–0.34)	-0.37 (-0.63–0.16)	<0.001
Maternal education, % (CI)			0.002
None	43.2 (37.7–48.8)	38.5 (32.4–45.0)	
Primary only	41.6 (36.3–47.1)	53.1 (46.9–59.2)	
Secondary or higher	15.2 (11.3–20.3)	8.5 (5.0–13.9)	
Bassa language speaker, % (CI)	55.9 (48.3–63.2)	59.2 (50.9–67.1)	0.355
Distance from health facility, median km (IQR)	5.6 (2.0–12.4)	6.1 (4.2–12.4)	0.313
Maternal age at birth, median (IQR)	26 (21–31)	25 (21–31)	0.734
Mother is married, % (CI)	79.9 (75.8–83.5)	78.7 (69.3–85.7)	0.715
Birth order			0.583
1st	39.0 (34.3–43.9)	35.3 (27.9–43.4)	
2nd or 3rd	38.4 (33.7–43.3)	39.1 (32.4–46.2)	
4th or higher	22.6 (19.3–26.4)	25.7 (19.0–33.7)	
Rainy season birth	45.8 (41.6–50.0)	45.6 (40.0–51.4)	0.970

*Household wealth index calculated with a principal components analysis including household asset ownership, floor, roof, and wall type, sanitation facilities, and water source, rescaled by dividing by its IQR.

**Fig 1:**
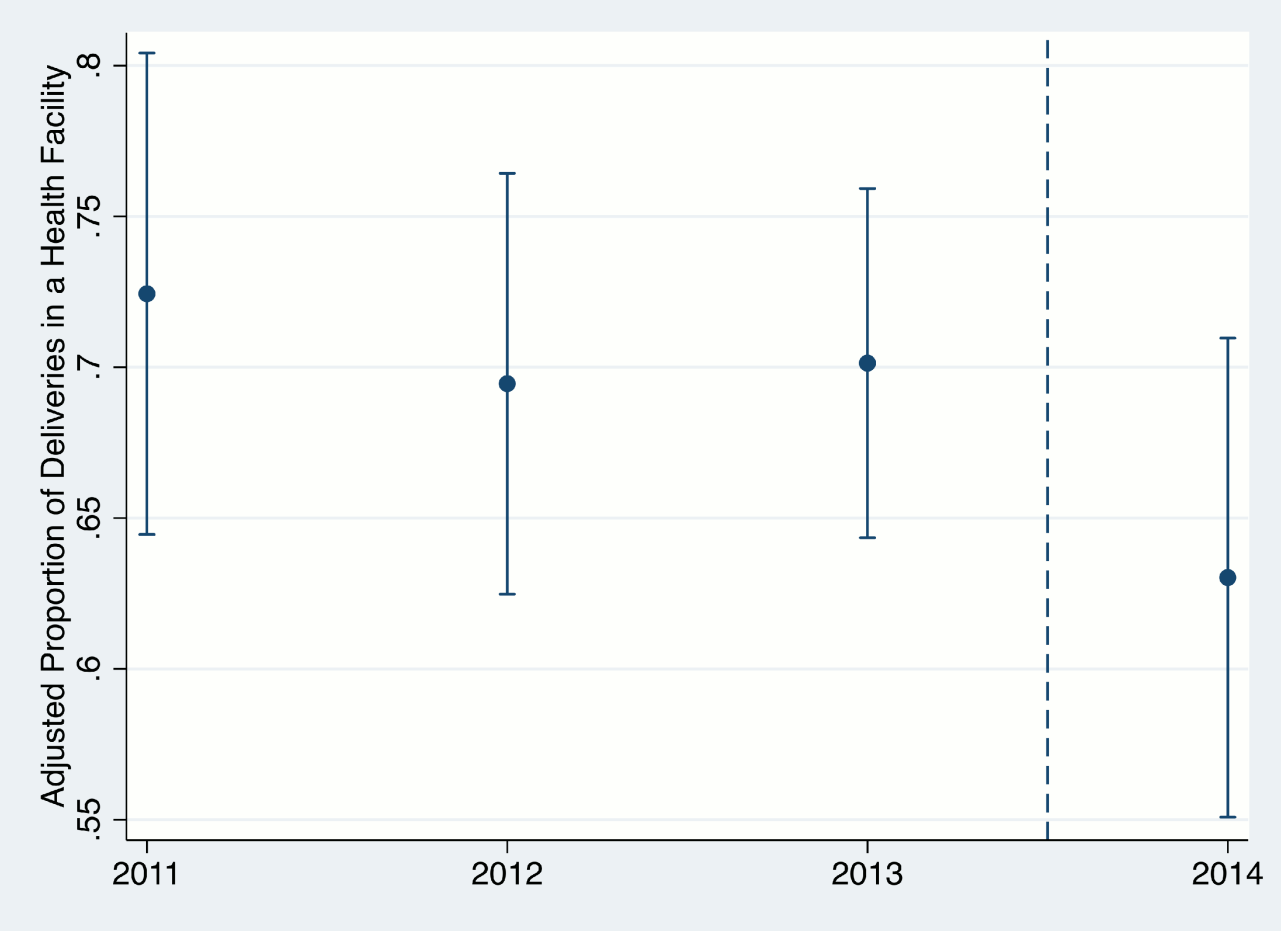
Proportion of women with facility-based delivery in Rivercess County, Liberia, during 2011–2014. Years begin in June, such that the 2014 estimate corresponds to the generalized Ebola virus disease epidemic in the country (June 2014–April 2015). Error bars represent 95% confidence intervals.

In the unadjusted analysis, birth during the Ebola period was associated with a 34% reduction in the odds of FBD (Table 2; OR = 0.66, 95% CI 0.48–0.90, *p* = 0.010). After adjustment for household wealth and maternal education, the odds of FBD were 30% lower during the Ebola period (AOR = 0.70, 95% CI 0.50–0.98, *p* = 0.037). This corresponds to a decrease in the adjusted FBD rate from 70.4% (95% CI 66.4–74.4%) in the pre-Ebola period to 62.9% (95% CI 54.9–70.8%) during the EVD epidemic (See Fig 1). These results did not change meaningfully after addition of other covariates (Table 2).

**Table 2:** Logistic regression models of facility-based delivery among women of child-bearing age in Rivercess County, Liberia, 2011–2015.

	Bivariate Associations		Multivariable Model 1		Multivariable Model 2		Multivariable Model 3	
Characteristic	OR (95% CI)	*p*-value	AOR (95% CI)	*p*-value	AOR (95% CI)	*p*-value	AOR (95% CI)	*p*-value
**Ebola period** *	**0.66 (0.48–0.90)**	**0.010**	**0.70 (0.50–0.98)**	**0.037**	**0.69 (0.49–0.97)**	**0.035**	**0.69 (0.50–0.97)**	**0.033**
Household wealth	1.77 (1.35–2.30)	<0.001	1.67 (1.29–2.17)	<0.001	1.25 (0.98–1.58)	0.069	1.26 (0.99–1.59)	0.062
Maternal education								
None	Ref.	Ref.	Ref.	Ref.	Ref.	Ref.	Ref.	Ref.
Primary only	1.20 (0.82–1.77)	0.348	1.19 (0.81–1.74)	0.379	1.09 (0.76–1.57)	0.626	1.05 (0.72–1.55)	0.782
Secondary or higher	2.40 (1.37–4.20)	0.003	1.44 (0.80–2.59)	0.224	1.54 (0.84–2.82)	0.160	1.53 (0.80–2.92)	0.193
Bassa language speaker	0.59 (0.38–0.90)	0.017			0.77 (0.50–1.17)	0.220	0.76 (0.49–1.18)	0.211
Distance from facility								
Per km, <10 km	0.81 (0.76–0.87)	<0.001			0.85 (0.78–0.92)	<0.001	0.85 (0.78–0.92)	<0.001
Per km, 10 to 21 km	1.03 (0.95–1.12)	0.410			1.00 (0.93–1.08)	0.993	1.00 (0.93–1.08)	0.966
Per km, >21 km	0.91 (0.81–1.02)	0.099			0.91 (0.82–1.00)	0.055	0.91 (0.83–1.01)	0.072
Maternal age at birth								
First quartile	Ref.	Ref.					Ref.	Ref.
Second quartile	0.74 (0.48–1.15)	0.176					0.73 (0.46–1.17)	0.192
Third quartile	0.69 (0.47–1.00)	0.050					0.71 (0.48–1.07)	0.100
Fourth quartile	0.75 (0.51–1.11)	0.145					0.75 (0.47–1.18)	0.208
Mother is married	0.83 (0.54–1.29)	0.410					1.04 (0.63–1.70)	0.882
Birth order								
1st	Ref.	Ref.					Ref.	Ref.
2nd or 3rd	0.84 (0.58–1.21)	0.340					0.89 (0.62–1.28)	0.514
4th or higher	0.92 (0.64–1.33)	0.652					1.16 (0.78–1.71)	0.454
Rainy season birth	0.89 (0.66–1.20)	0.445					0.87 (0.63–1.19)	0.371

*Pre-Ebola virus disease period defined as March 2011–June 14, 2014; Ebola virus disease period defined as June 15, 2014–April 2015.

Belief that health facilities posed a risk of EVD transmission was reported by 56.0% (95% CI 51.5–60.4) of respondents. Adjusting for the same potential confounders as in multivariable model 1, the odds of FBD were 41% lower (AOR = 0.59, 95% CI 0.36–0.97, *p* = 0.038) during the EVD epidemic then prior among women who reported believing that health facilities are or may be a source of Ebola transmission. In contrast, no significant difference was detected between time periods among those who did not report such a belief (AOR = 0.90, 95% CI 0.59–1.37, *p* = 0.612). Unadjusted and adjusted results are presented in S1 and S2 Tables.

The observed association between delivery during the Ebola period and FBD varied little in the sensitivity analyses (S2–S14 Tables). Observed odds ratio point estimates remained between 0.69 and 0.79 and significant or marginally significant in all analyses but one. The one exception was an analysis that selected August 6 as the start of the Ebola period (S13 Table), for which the relationship lost statistical significance (AOR = 0.79, 95% CI 0.56–1.12, *p* = 0.186). In this same conceptualization, birth during the Ebola period remained significantly associated with lower FBD among those who believed health facilities to be an Ebola transmission source (AOR = 0.60, 95% CI 0.36–0.99, *p* = 0.044) but not among those who did not report this belief (OR = 1.03, 95% CI 0.63–1.70, *p* = 0.902).

## Discussion

This study is the first to our knowledge to leverage population-based survey data to examine relationships between the West African Ebola epidemic and facility-based delivery. Importantly, our survey was conducted in a county of Liberia with relatively few confirmed Ebola cases and in which health facilities never officially closed. We estimate a 30% reduction in the odds of facility-based delivery during the Ebola period in this area of the country. This corresponds to an approximately 8-percentage-point reduction in FBD, which is substantial in a country where FBD had only increased by 20 percentage points since the first post-war survey in 2007 [27,28]. If the same reduction in FBD we identified were extrapolated to all of rural Liberia, we would estimate approximately 5,900 deliveries would occur outside health facilities because of fear or other Ebola-related barriers to facility-based delivery (see S2 Appendix). However, this figure might substantially underestimate the impact in certain high-burden areas, where the Ebola epidemic resulted in facility closures. We observed the reduction in FBD to only be significant among those who perceived health facilities to be a possible or definite risk for EVD transmission, suggesting that Ebola-related fear was a major factor in the decrease in health utilization.

Our results are in keeping with prior assessments of the collateral effects of the West African Ebola epidemic on non-Ebola health outcomes. One modeling study by Takahashi et al. estimated that vaccine disruptions may be sufficiently large to undermine population immunity against measles and other childhood illnesses [10]. Another model by Parpia and colleagues suggests that excess deaths from interruptions to HIV/AIDS, tuberculosis, and malaria programs may rival direct Ebola deaths in the three most affected countries [22]. Walker et al.’s model suggested that treatment interruptions would increase the number of untreated malaria cases by 3.5 million in 2014 [11].

Studies based on health management information systems (HMIS) data have also suggested deleterious health consequences. Two studies of maternal health visits in heavily affected Liberian counties where facilities closed or non-essential services were restricted found reductions of approximately 80% in recorded services [7,20]. Hospital record studies have shown similar reductions in maternal health utilization in Guinea [12] and lesser but substantial reductions in Sierra Leone [21]. Others found substantial reductions in surgical services and outpatient services [13,19]. A Guinean study found a 42% increase in HIV treatment default at the country’s largest treatment site [14], and a Liberian study similarly found a 41% reduction in HIV clinic visits from May to June at a Monrovia hospital in which a healthcare worker was infected in June [15]. Another Liberian study found significant degradation in the total number of visits, number of new patients, and delays for follow-up among HIV patients at two hospitals in Monrovia [16]. Guinean reviews of clinic records found comparable reductions in HIV testing and enrollment, tuberculosis diagnoses, and outpatient care in one study [17] and substantial reductions in malaria treatment in another [18].

Our study is unique from prior reports in the use of population-based survey data, which provides two important methodological benefits. First, our study design, using community-based, population-representative sampling, enables population-wide estimation and reduces selection bias from clinic-based samples. Second, our analysis avoids confounding, which might occur in health records and HMIS-based studies, in which timely, accurate, and/or complete recordkeeping interruptions might also be impacted by public health shocks.

Because our study is observational, causality can be inferred but not proven [69,70]. Causal inference in our study is supported by two factors. First, the Ebola epidemic was exogenously introduced and therefore unrelated to secular trends in the Liberian health system. Second, a stratified analysis demonstrated decreased FBD during the Ebola period, principally among women reporting a belief that health facilities were potential EVD transmission sites. The principal threat to causality is secular changes coincident with the Ebola epidemic, which independently reduced FBD. However, this risk is mitigated by several aspects of our study, including Ebola’s exogeneity. Furthermore, FBD increased in Liberia in the years preceding the Ebola epidemic [27,28], so secular trends would be expected to bias our results toward the null. Finally, our findings are robust to sensitivity analyses, including variations in the EVD start date and definitions of inclusion criteria.

Our study is also subject to the standard limitations of population-based surveys, including potential selection and non-response biases, recall errors, and response biases [71,72]. We mitigated selection bias by enumerating villages immediately prior to sampling, and our response rates were within customary limits for demographic and health surveys [73]. We limited response biases by utilizing broadly accepted demographic and health survey items. Recall errors cannot be excluded, but the principal threat to our findings would be if women who delivered at home erroneously reported delivering in a health facility during the pre-Ebola period but not during the Ebola period, and if countervailing error did not occur among women who delivered in a facility. Finally, we cannot exclude the possibility of social desirability bias, which could affect FBD reporting in either direction: over the long term, it most likely causes over-reporting, as Liberia has prioritized FBD, but there is a chance of underreporting if facility visits during the epidemic were stigmatized. This risk should be mitigated, because virtually all women reported deliveries older than Ebola’s incubation period (and, therefore, possibility of infection).

Our findings should be generalizable to similar regions of the three principally affected countries. In Liberia, most of the southern and eastern parts of the country had sporadic cases, as was seen in the catchment area studied here. This was also the case in many parts of rural Guinea and, to a lesser degree, Sierra Leone [1]. Several risk perception surveys and qualitative analyses have documented substantial fears about acquiring Ebola from health facilities in Sierra Leone and Liberia [65,74,75]. In contrast, our data are less likely to represent effects in regions with differing epidemic burdens. For example, one would expect greater FBD reductions in areas with a greater EVD caseload, areas with facility closures, and/or areas with decreased availability of healthcare workers or supplies, such as in Monrovia, where routine health services largely ceased during the epidemic’s peak. In such areas, our results likely underestimate the total collateral effects of the epidemic. Future work to document health disruptions through population-based surveys in a wider variety of locations would be valuable, as would additional modeling studies that incorporate greater subnational heterogeneity in epidemic intensity.

Our findings have implications for countries with ongoing Ebola epidemics and for post-Ebola public health initiatives. Though health systems were most drastically affected in locations where Ebola transmission directly affected healthcare workers and facilities, our results suggest that significant collateral health effects also occurred in relatively lightly affected regions. This finding reinforces a need to strengthen and maintain basic services amidst public health emergencies, and also that risk communication strategies to prevent and curtail stigma are vital to maintaining demand for health services. Our results also add support to recent calls from within West Africa and the greater global health community to invest in health systems strengthening in the region [26,76,77]. Both our results and those of prior studies reinforce that the need will extend beyond epidemiologic surveillance and EVD vaccination to include investments in basic healthcare delivery, even in areas distant from the epicenters of the epidemic.

Indeed, efforts to maintain basic, primary health care should be considered an essential part of the response to outbreaks and other emergencies. Anecdotal reports from affected locations include the interruption of a wide range of essential services—from family planning outreach to fever care and community vaccination campaigns—even in communities where facility-based services continued. While building and staffing Ebola treatment units was a critical part of the public health response, in future epidemics it is important to incorporate efforts to maintain staffing and services to address routine health needs. Additionally, delegating services to community health workers or other community-based providers may enable continuity of routine services while also providing inroads into communities that can strengthen epidemic surveillance and response [78–82]. Finally, though our analysis examines collateral healthcare consequences, the epidemic also appears to have exacerbated social vulnerability, including worsened poverty, the loss of a year of education, increased child labor, and broad psychosocial consequences [83]. Increasing resiliency to epidemics and other emergencies should be a top global priority [84].

In the immediate term, health ministries in Ebola-affected locations and their partners should redouble efforts to restore FBD and other health service utilization to pre-Ebola levels and to continue positive trajectories achieved before the epidemic. For instance, the Liberian Ministry of Health’s Post-Ebola Investment Plan calls for not only strengthening epidemic surveillance and control efforts but also achieving universal health coverage—including the creation of a National Health Workforce Program that will lead to the deployment of thousands of new primary health care workers, including rural nurses, midwives, and community health workers. Such strategies will require a restoration of trust and amelioration of fear of health facilities [65]. Active engagement of community leaders; effective, culturally appropriate, and evidence-based communication strategies; and involvement of trusted local health and lay figures will likely be required as affected countries rebuild their health sectors [4,85].

## Supporting Information

S1 Appendix Smoothed graph of facility-based delivery as a function of a continuous date of birth variable. (DOC)

S2 Appendix Estimate of the reduction in rural Liberian births occurring in health facilities (not including facility closures). (XLSX)

S1 Checklist STROBE checklist. (DOC)

S1 Code Replication statistical code (Stata format). (DO)

S1 Dataset Replication dataset in Stata format. (DTA)

S2 Dataset Replication dataset in Excel format. (XLSX)

S1 Fig LOWESS curves of facility-based delivery by distance and wealth. (EPS)

S1 Survey Survey instrument. (DOCX)

S1 Table Analysis restricted only to those respondents who reported a belief that clinics did or may pose an Ebola transmission risk. (DOCX)

S2 Table Analysis restricted only to those respondents who reported a belief that clinics did not pose an Ebola transmission risk. (DOCX)

S3 Table Sensitivity analysis: restricted to only households who lived in current village at the time of the delivery. (DOC)

S4 Table Sensitivity analysis: excludes births within 2 wk before the survey began. (DOC)

S5 Table Sensitivity analysis: excludes women over age 45 at time of birth to avoid possible bias from attrition. (DOC)

S6 Table Sensitivity analysis: excludes births within 2 wk of the survey, women over age 45 at the time of delivery, and women who lived in a different village at the time of delivery. (DOC)

S7 Table Sensitivity analysis: includes observations from 2012 to 2015. (DOC)

S8 Table Sensitivity analysis: includes observations from 2013 to 2015. (DOC)

S9 Table Sensitivity analysis: includes observations from 2010 to 2015. (DOC)

S10 Table Sensitivity analysis: jackknifed standard errors instead of Taylor linearization. (DOC)

S11 Table Sensitivity analysis: Ebola period begins on May 29, 2014, when Liberia’s second wave began. (DOC)

S12 Table Sensitivity analysis: Ebola period begins on July 15, 2014, by which point Ebola was a national epidemic. (DOC)

S13 Table Sensitivity analysis: Ebola period begins on August 6, 2014, when Liberia’s president declared a national emergency. (DOC)

S14 Table Sensitivity analysis: random redistribution of heaped birth dates from first day of month. (DOC)

S1 Text Analysis history for the analysis reported in this study. (DOCX)

## Abbreviations

CHW: community health worker
DHS: demographic and health surveys
EVD: Ebola virus disease
FBD: facility-based delivery
GPS: global positioning system
HMIS: health management information systems
LOWESS: locally weighted scatterplot smoothing
