# Supplementary material for: Facility-Based Delivery during the Ebola Virus Disease Epidemic in Rural Liberia: Analysis from a Cross-Sectional, Population-Based Household Survey
Source: PLoS Med. 2016 Aug 2;13(8):e1002096. doi: 10.1371/journal.pmed.1002096 (PMC4970816; doi:10.1371/journal.pmed.1002096)
Supplement: S1 Appendix — (DOC) [file pmed.1002096.s001.doc]

**Appendix 1: Smoothed Graph of Facility-based Delivery over Continuous Time**


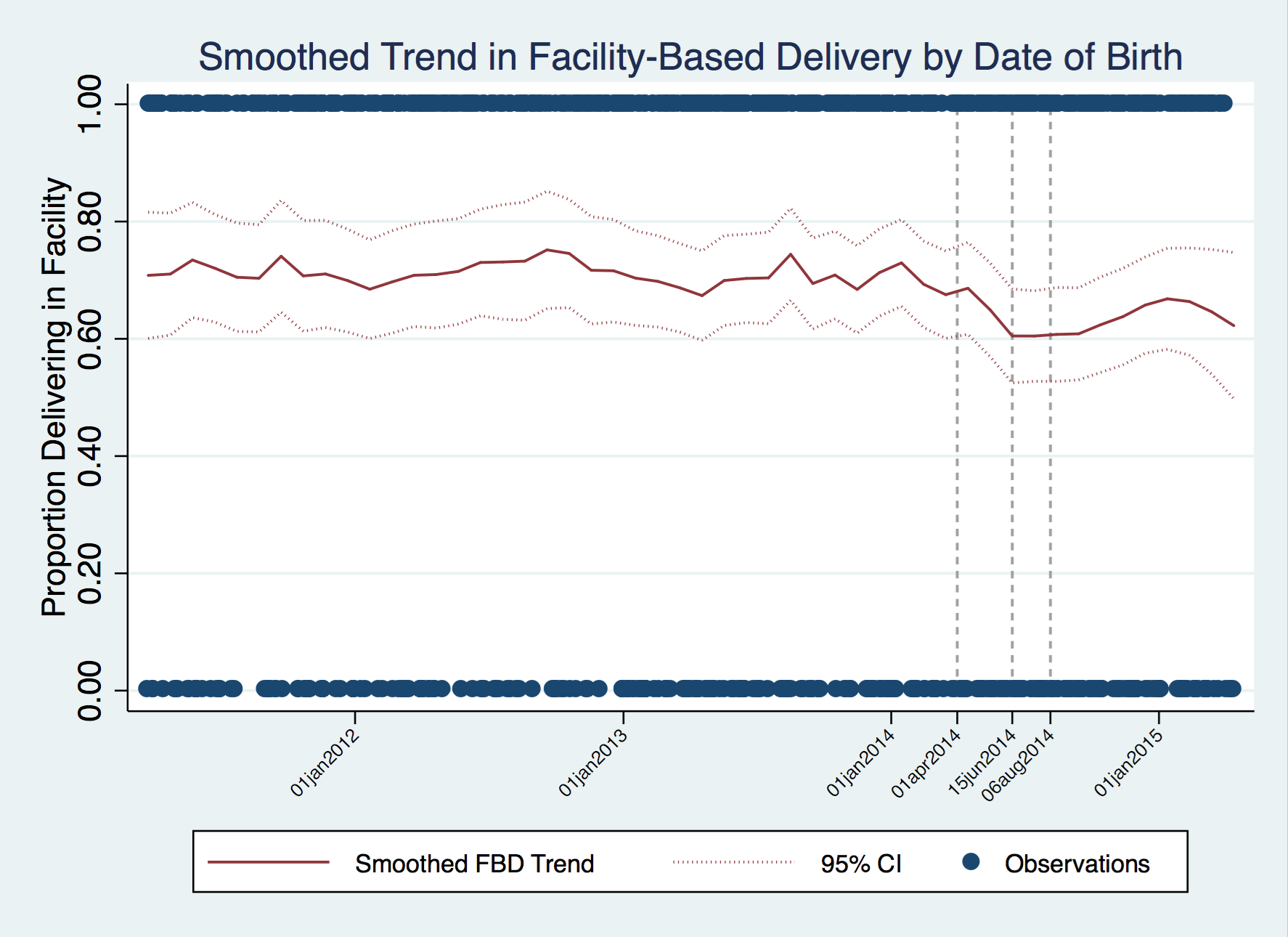


Methods:

We plotted the proportion of births in a health facility using local polynomial smoothing with a rectangular kernel (bandwidth=100). We applied analytical weights, which are a permissible substitution for sampling weights because weights were not associated with time period of delivery, to represent population-level trend over time, but confidence intervals should be treated as approximate. CIs are likely slightly conservative because this survey has a low design effect because a relatively high fraction of the population was sampled and because of gains in efficiency due to stratification (DEFT for the main analysis=0.96).

Reference lines represent potentially critical dates: April 1, 2014, when the epidemic was initially recognized; June 15, 2014, which designates the Ebola period in this paper; and August 6, 2014, when Liberia declared an national emergency due to Ebola.
