# Supplementary material for: Facility-Based Delivery during the Ebola Virus Disease Epidemic in Rural Liberia: Analysis from a Cross-Sectional, Population-Based Household Survey
Source: PLoS Med. 2016 Aug 2;13(8):e1002096. doi: 10.1371/journal.pmed.1002096 (PMC4970816; doi:10.1371/journal.pmed.1002096)
Supplement: S1 Survey — (DOCX) [file pmed.1002096.s008.docx]

**LAST MILE SURVEY – Household Questionnaire (page 1)** Svy ID: _______________________________

Village name: ____________________ Household ID#: _____ Today’s date(d/m/y):_______________________

Survey language: __ English __ Krahn __ Bassa __ French __ Other : __________

SIGN HERE IF INFORMED CONSENT WAS OBTAINED FROM THE FEMALE HEAD OF HOUSEHOLD: _____________________

ASK THE QUESTIONS IN SECTION 1 TO THE FEMALE HEAD OF HOUSEHOLD; ONLY FILL OUT ONE PER HOUSEHOLD

1. HOUSEHOLD QUESTIONS

­­­­­1.1 Please give me the first names of the persons who are living in your house together, with even strangers and visitors who slept here last night. Please start from the land-lady or land-lord.

| # | First name | Sex | Age  (or birth year) | (NAME) living here all the time? | (NAME) slept here last night? | Eligible? |
| --- | --- | --- | --- | --- | --- | --- |
| 1 |  | __Male  __Female |  | __Yes  __No | __Yes  __No | __Eligible  __ Not eligible |
| 2 |  | __Male  __Female |  | __Yes  __No | __Yes  __No | __Eligible  __ Not eligible |
| 3 |  | __Male  __Female |  | __Yes  __No | __Yes  __No | __Eligible  __ Not eligible |
| 4 |  | __Male  __Female |  | __Yes  __No | __Yes  __No | __Eligible  __ Not eligible |
| 5 |  | __Male  __Female |  | __Yes  __No | __Yes  __No | __Eligible  __ Not eligible |
| 6 |  | __Male  __Female |  | __Yes  __No | __Yes  __No | __Eligible  __ Not eligible |
| 7 |  | __Male  __Female |  | __Yes  __No | __Yes  __No | __Eligible  __ Not eligible |
| 8 |  | __Male  __Female |  | __Yes  __No | __Yes  __No | __Eligible  __ Not eligible |
| 9 |  | __Male  __Female |  | __Yes  __No | __Yes  __No | __Eligible  __ Not eligible |
| 10 |  | __Male  __Female |  | __Yes  __No | __Yes  __No | __Eligible  __ Not eligible |

1.2: Mark “Eligible” in last column if household member is a woman age 18-49 who slept in house last night.

1. Is there anyone in this house who get farm land? __ Yes

__ No

1. Where do you people get water from for drinking? __ hand pump, protected well __ Tanker truck

__ Rainwater __ public tap/standpipe

__ Bottled water __ Piped into dwelling

__ Surface water (creek/pond/canal) __ Protected spring

__ tube well or borehole __ Piped to yard/plot

__ Unprotected well __ Cart with small tank

__ Unprotected spring __ Other

**LAST MILE SURVEY – Household Questionnaire (page 2)**

1. Where do you people go to toilet/poo poo? __ Pit latrine without slab/open pit __ Hanging toilet/hanging latrine

__ Pit latrine with slab __ Flush to pit latrine

__ Ventilated improved pit latrine __ Flush to septic tank

__ No facility/bush/field __ Flush to piped sewer system

__ Composting toilet __ Flush to somewhere else

__ Bucket toilet __ Other

__ Flush, don’t know where

1. People from other houses go to the same toilet? __Yes

__ No

1. Does your household have: [READ ALL] __ Chairs? __ Ice box?

__ Table? __ Electricity?

__ Cell phone? __ Solar panel?

__ Radio? __ Generator?

__ Cupboard? __ Television?

__ Mattress (not grass)? __ Computer?

__ Sewing machine? __(check here if none)

__________________________________________________________________________________________________________________________________________________

1. What you use for cooking – that coal, gas stove, __ Wood __ Kerosene stove

wood, other thing? __ Fire coal/charcoal __ Biogas

__ Gas cylinder __ Electricity

__ No food cooked in house __ Other

1. RECORD THE MAIN MATERIAL OF __ Earth/sand/mud __ Wood planks

THE FLOOR OF THE HOUSE __ Concrete, cement __ Carpet

__ Floormat, linoleum, vinyl __Parquet or polished wood

__ Ceramic tiles __ Other

1. RECORD THE MAIN MATERIAL OF __ Thatch/palm leaf __ Wood

THE ROOF OF THE HOUSE __ Tarpaulin, plastic __ Rustic mat

__ Zinc, metal __ Ceramic tiles

__ Palm/bamboo __ Asbestos sheets, shingles

__ Wood planks __ Other

__ Concrete, cement

1. RECORD THE MAIN MATERIAL OF __ Cane/palm/trunks __ Reused wood

THE OUTSIDE WALLS OF THE HOUSE __ Mud and sticks __ Cement

__ Stone blocks __ Wood planks/shingles

__ Mud bricks __ Straw, thatch mats

__ Bricks __ Cardboard, plastic

__ Plywood __ Zinc, metal

__ Other

1. Anyone in this house here get: [READ ALL] __ a watch? __ a car/truck?

__ a bicycle? __ a boat/canoe?

__ a motorbike? __ (check here if none)

1. You have any animals, chickens, or ducks in this house? __Yes

__ No 🡪 1.15

1. How many animals the people in this house get, like… [READ ALL] Cows: ___ Goats: ____ Sheep: ____

Pigs: ___ Chicken, ducks, birds: ____

1. Anyone in this house who get money in the bank? __Yes

__ No

**LAST MILE SURVEY – Female Questionnaire (page 1)** Svy ID: ____________________________

Village name: ____________________ Household ID#: _____ Today’s date(d/m/y):___________________

Survey language: __ English __ Krahn __ Bassa __ French __ Other : __________

SIGN HERE IF INFORMED CONSENT WAS OBTAINED FROM THE RESPONDENT: _____________________

ASK THE QUESTIONS IN SECTION 1 TO THE FEMALE HEAD OF HOUSEHOLD; ONLY FILL OUT ONE PER HOUSEHOLD

**2. MATERNAL HEALTH + FAMILY PLANNING + CHILD MORTALITY**

- - - 1. What year you were born? ________ ___age

___ birth year

- - - 1. How long you been living here in this village? ___ years ___ months ___always ___just visiting

(Probe: You been living here the whole time?)

- - - 1. You been to school before? Which class you stop in? ___ high school graduate ___ primary school

___ some high school ___ no school

- - - 1. From five years up to now, you ever born a child? ___yes 🡪 2.5 ___ no 🡪 2.24
      2. When was your last birth? _________ month _______ year 🡪 If before APRIL 2010 then skip to 2.24
      3. Did you see anyone for big belly checkups when you had belly with that child? ___yes 🡪 2.7

___ no 🡪 2.10

- - - 1. Who did you see for big belly checkups? [MORE THAN ONE] ___ doctor ___physician assistant (PA)

Any other person? ___ nurse ___ certified midwife

___ gCHV __ country midwife/Zoe

___ FHW ___ trained traditional midwife (TTM)

___ other: __________________

- - - 1. Where did you go to get the checkups? Any other place? ___ home ___ other home ___ drug store

___ clinic/hospital ___ other: ________

__________________________________________________________________________________________________________________________________________________

- - - 1. How many times did you get big belly checkups when you had belly with that child? _____(number)
      2. Where you born the child? ___ Clinic/hospital 🡪 2.11

___ Your home 🡪 2.15

___ Other home 🡪 2.15 ___ Other: _____ 🡪 2.15

__________________________________________________________________________________________________________________________________________________

- - - 1. How long you stayed in the clinic after you born the child? ________ ___hours __ days __weeks __don’t know
      2. After the child was born but before you left the clinic, any ___yes 🡪 2.13

doctor or nurse check you to know how you were coming on? ___no 🡪 2.18

- - - 1. How long it took after you born the baby before they first check on you? ________ ___hours __ days __weeks __don’t know
      2. Who check on you to see how you were coming on that time? Any other person? [MORE THAN ONE]__ Clinic staff 🡪 2.18 ___ TTM 🡪 2.18

___ country midwife/Zoe 🡪 2.18

___ Other: ____ 🡪 2.18

**LAST MILE SURVEY – Female Questionnaire** **(page 2)**

- - - 1. After you born the child, any person check on you to see __yes 🡪 2.16

how you coming on that time? __ no 🡪 2.18

- - - 1. How long it took after you born the baby before they first check you? ________ ___hours __ days __weeks ___ don’t know
      2. Who check you to see how you were coming on that time? [MORE THAN ONE] __ clinic staff __ gCHV __TTM __ FHW

__country midwife/Zoe __ Family/friends __Other: _______

- - - 1. The child you born, is it still alive? __yes 🡪 2.20

___ no 🡪 2.19

- - - 1. When the child die? ___ at birth 🡪 2.24

___ days 🡪 2.20

___ months 🡪 2.20

___ years 🡪 2.20

- - - 1. Any person check on the baby condition __ yes 🡪 2.21

in the first two months after the child was born? __ no 🡪 2.24

__ don’t know 🡪 2.24

- - - 1. How many hours, days, or weeks it took after you born ________ ___hours __ days __weeks ___ don’t know

the baby before they first check on his/her condition?

- - - 1. Who check on the baby condition at that time? [MORE THAN ONE] __ clinic staff __ gCHV __TTM ___ FHW __Country midwife/Zoe __ Family/friends __Other: _______
      2. What place they check on the baby first? __ clinic/hospital __ your home __ other home __other: ____________

**LAST MILE SURVEY – Female Questionnaire** **(page 3)**

- - - 1. Now I will like to talk to you about family planning. You marry __yes 🡪 2.26

Now-now or you living with a man just like you people marry? __ no 🡪 2.25

- - - 1. You ever had something to do with man in this month that passed? __yes 🡪 2.26

__ no 🡪 2.39

- - - 1. You currently doing something or using any method __yes 🡪 2.27

to delay or avoid getting pregnant? __ no 🡪 2.28

__ I can’t get pregnant 🡪 2.39

- - - 1. Which thing you using? [MORE THAN ONE] __ pill __ condom __ IUD __ Rhythm method

__ injection __ withdrawal __ withdrawal

__ emergency contraception __other: __________

- - - 1. You get belly now-now? __yes 🡪 2.32

__ no 🡪 2.29

__ Don’t know 🡪 2.29

__ I can’t get pregnant 🡪 2.39

- - - 1. You born a child in the last two years? __yes 🡪 2.30

__ no 🡪 2.33

__ I can’t get pregnant 🡪 2.39

- - - 1. You now start seeing your time since your last belly? __yes

__ no

- - - 1. The last time you get belly, you wanted it that time? __yes 🡪 2.33

__ no 🡪 2.33

- - - 1. This belly you get now-now, the time it came you wanted it? __yes 🡪 2.34

__ no 🡪 2.34

- - - 1. You still want some more children? __yes 🡪 2.34

__ no 🡪 2.35

__ I can’t get pregnant 🡪 2.39

- - - 1. You want to have your next child some time in the next two years? __yes

__ no

__ I don’t know

- - - 1. You know any place where a person can get some for family planning? __yes 🡪 2.36

__ no 🡪 2.39

__ I don’t know 🡪 2.39

- - - 1. Where is that? Any other place? [MORE THAN ONE] __ drug store __ hospital/clinic __ gCHV __FHW

__ tablet man/black bagger __other: _________

- - - 1. If you wanted to, could you yourself get something for family planning? __yes

__ no

__ I don’t know

- - - 1. You stop seeing your time at all? __yes, I stopped seeing my time

__ no , I still seeing my time

__ I don’t know

- - - 1. It not that I want to know how it was looking, but you ever go to do __yes

your HIV/AIDS test before? __ no

- - - 1. Have you been seen by a community health worker in the last 3 months? __yes 🡪 2.41

__ no

- - - 1. Which one? Any other person? (MORE THAN ONE) __gCHV __FHW

__TTM __other: __________

___ country doctor

**LAST MILE SURVEY – Female Questionnaire** **(page 4)**

- - - 1. Now I want the first names of all the children you born, whether still alive or not, starting with the first one.

IF AGE AT DEATH IS 1 YEAR, ASK “how many months?”RECORD DAYS IF LESS THAN 1 MONTH.MONTHS IF LESS THAN 2 YEARS

| 2.43: What is the first name of your (first/next) child? | 2.44: Is (NAME) a boy of a girl? | 2.45: In what month and year was (NAME) born? | 2.46: Was (NAME) born in a clinic? | 2.47: Is (NAME) still living? | 2.48: IF DEAD, how old was (NAME) when he/she died? |
| --- | --- | --- | --- | --- | --- |
| 1 | _ Boy  _ Girl | Year: _____  Mth: ______  Day: ______ | __Yes  __ No | __Yes  __ No | ____ __years  __ mths  __ days |
| 2 | _ Boy  _ Girl | Year: _____  Mth: ______  Day: ______ | __Yes  __ No | __Yes  __ No | ____ __years  __ mths  __ days |
| 3 | _ Boy  _ Girl | Year: _____  Mth: ______  Day: ______ | __Yes  __ No | __Yes  __ No | ____ __years  __ mths  __ days |
| 4 | _ Boy  _ Girl | Year: _____  Mth: ______  Day: ______ | __Yes  __ No | __Yes  __ No | ____ __years  __ mths  __ days |
| 5 | _ Boy  _ Girl | Year: _____  Mth: ______  Day: ______ | __Yes  __ No | __Yes  __ No | ____ __years  __ mths  __ days |
| 6 | _ Boy  _ Girl | Year: _____  Mth: ______  Day: ______ | __Yes  __ No | __Yes  __ No | ____ __years  __ mths  __ days |
| 7 | _ Boy  _ Girl | Year: _____  Mth: ______  Day: ______ | __Yes  __ No | __Yes  __ No | ____ __years  __ mths  __ days |
| 8 | _ Boy  _ Girl | Year: _____  Mth: ______  Day: ______ | __Yes  __ No | __Yes  __ No | ____ __years  __ mths  __ days |
| 9 | _ Boy  _ Girl | Year: _____  Mth: ______  Day: ______ | __Yes  __ No | __Yes  __ No | ____ __years  __ mths  __ days |
| 10 | _ Boy  _ Girl | Year: _____  Mth: ______  Day: ______ | __Yes  __ No | __Yes  __ No | ____ __years  __ mths  __ days |

QUESTIONS 2.49 – 2.52 ARE ABOUT THE WOMAN’S MOST RECENT BIRTH, WHETHER DEAD OR ALIVE.

1. NAME OF MOST RECENT BIRTH: ______________________

If child reached 6 months (alive or dead)

1. Before (NAME) reach 6 months, that only titi water you give, or you give thing like juice or creek water? __ baby had only titi __ baby had other things

If child did not reach 6 months (alive or dead)

1. That only titi water you give, or you sometimes give thing like juice or creek water? ____ baby had only titi

| 1 | 2 | 3 |
| --- | --- | --- |
| 4 | 5 | 6 |

__ baby had other things

1. WRITE DOWN NAME OF EVERY

CHILD BORN IN APRIL 2010 OR LATER

**LAST MILE SURVEY – Child Questionnaire** **(page 1)** Svy ID: ____________________

Village name: ____________________ Household ID#: _____ Today’s date(d/m/y):___________________

Survey language: __ English __ Krahn __ Bassa __ French __ Other : __________

ASK THESE QUESTIONS FOR EACH CHILD LESS THAN 5 THAT THE WOMAN GAVE BIRTH TO.

**3. CHILD HEALTH**

1. WRITE DOWN THE CHILD’S NAME AND NUMBER FROM 2.64: Number _____ Name: __________________
2. (NAME) ever had running stomach in these two weeks that passed? __ yes 🡪 3.2

__ no 🡪 3.9

__ don’t know 🡪 3.9

1. You got treatment from anywhere or anyone the time __ yes 🡪 3.4

(NAME) stomach was running? __ no 🡪 3.9

1. Where or who (NAME) got treatment from? Any other place? [MORE THAN ONE] __ drug store __ tablet man/black bagger

___ gCHV __ hospital/clinic __other: ______

___ country doctor __Zoe __ FHW

1. Who did you go to for treatment or advice **FIRST?** [ONLY ONE] ___ drug store __ tablet man/black bagger

___ gCHV __ hospital/clinic __other: ______

___ country doctor __Zoe ___ FHW

1. When the stomach started running, how many days it took before __________ (days) __don’t know

(NAME) get treatment?

1. Since (NAME) stomach started running, anybody give him/her ___yes __ no ___ don’t know

Glucose water (ORS) to drink?

1. Since (NAME) stomach started running, anybody give him/her ___yes __ no ___ don’t know

Homemade sugar/salt drink?

1. (NAME) ever got hot (fever) in these two weeks that passed? __ yes 🡪 3.10

__ no 🡪 3.12

__ don’t know 🡪 3.12

1. When (NAME) skin was hot, anyone take blood __ yes 🡪 3.11

From his/her finger or heel to do malaria test? __ no 🡪 3.12

__ don’t know 🡪 3.12

1. (NAME) test showed that he/she got malaria? ___yes __ no ___ don’t know
2. (NAME) ever got cough in these two weeks that passed? __ yes 🡪 3.13

__ no 🡪 3.15

__ don’t know 🡪 3.15

1. The time (NAME) was coughing, you saw him/her breathing __ yes 🡪 3.14

Fast-fast or he/she was catching hard time to breathe? __ no 🡪 3.15

__ don’t know 🡪 3.15

1. You think the fast breathing was caused by some problem in __ chest only __ nose only __both chest and nose

[NAME] chest or something closing [NAME] nose, or both? __ don’t know __ other

1. IF CHILD HAD FEVER OR COUGH, ASK QUESTION 3.16; IF CHILD HAD NO ILLNESS, SKIP TO 3.21.
2. You got treatment from anywhere or anyone the time [NAME] was sick? __ yes 🡪 3.17

__ no 🡪 3.21

1. Where or who (NAME) got treatment from? [MORE THAN ONE] ___ drug store __ tablet man/black bagger

Any other place? ___ gCHV __ hospital/clinic __other: ______

___ country doctor __Zoe ___ FHW

1. Who did you go to for treatment **FIRST?** [ONLY ONE] ___ drug store __ tablet man/black bagger

___ gCHV __ hospital/clinic __other: ______

___ country doctor __Zoe ___FHW

_____________________________________________________________________________________________________________________________________________________

1. When the sickness started, how many days it took before (NAME) get treatment? _____ (days) __don’t know

**LAST MILE SURVEY – Child Questionnaire** **(page 2)**

1. What kind of medicine (NAME) took? Any other medicine? [MORE THAN ONE] __ new malaria tablet/ACT __chloroquine

__ country medicine __ antibiotics

__ don’t know __ other: ________________

1. (NAME) sleep under mosquito net last night? ___yes __ no ___ don’t know

FOR CHILDREN 2 AND UNDER

1. You get vaccine card for (NAME)? ___yes 🡪 3.23

___ no 🡪 3.27

1. I can please see it? ___yes 🡪 3.24

___ no 🡪 3.27

1. COPY DOWN ALL VACCINES RECEIVED, AND RECORD DATES.

| Vaccine: | | BCG | | Penta 1 | | Penta 2 | Penta 3 | Polio (P0) | Polio (P1) | Polio (P2) | Polio (P3) | Measles | | Yellow Fever |  |
| --- | --- | --- | --- | --- | --- | --- | --- | --- | --- | --- | --- | --- | --- | --- | --- |
| Received? | | __ | | __ | | __ | __ | __ | __ | __ | __ | __ | | __ |  |
| Date: | |  | |  | |  |  |  |  |  |  |  | |  |  |
|  |  | |  | |  | | | | | | | | | | |
| Vaccine: | | Rota 1 | | Rota 2 | | Rota 3 | Pneumo 1 | Pneumo 2 | Pneumo 3 | IPV 1 | IPV 2 | IPV 3 |  |  |  |
| Received? | |  | |  | |  |  |  |  |  |  |  |  |  |  |
| Date: | |  | |  | |  |  |  |  |  |  |  |  |  |  |

1. (NAME) ever take any other vaccine that they didn’t write __ yes 🡪 3.26

on the vaccine card? Like those ones they can give all over the __ no 🡪 4.1

country? __ don’t know 🡪 4.1

1. Which ones? [MORE THAN ONE] Vaccine received? 🡪 4.1

| BCG __ | Polio (P1) __ | Rota 3 __ | IPV 2 __ |
| --- | --- | --- | --- |
| Penta 1 __ | Polio (P2) __ | Pneumo 1 __ | IPV 3 __ |
| Penta 2 __ | Polio (P3) __ | Pneumo 2 __ | Measles __ |
| Penta 3 __ | Rota 1 __ | Pneumo 3 __ | Yellow fever |
| Polio (P0) __ | Rota 2 ___ | IPV 1 ___ |  |

__ Don’t know

1. (NAME) ever take any vaccine to prevent him/her from getting diseases, __ yes 🡪 3.28

at the clinic or like those ones they can give all over the country? __ no 🡪 4.1

__ don’t know 🡪 4.1

1. (NAME) ever take the TB vaccine that can make a mark on the child’s arm or shoulder? __ yes

__ no

__ don’t know

1. (NAME) ever take the vaccine for the sickness that can make children __ yes 🡪 3.30

cripple they call polio? That the vaccine they can drop in the child’s mouth. __ no 🡪 3.32

__ don’t know 🡪 3.32

1. How old was (NAME) when they give him/her the first vaccine in the ___ first two weeks

mouth? It was in the first two weeks after he/she was born or it was more than two weeks? ___ later

___ don’t know

1. How many times they now put the polio vaccine in (NAME) mouth since ___________ (number)

He/she was born? ___ don’t know

1. They ever give (NAME) the pentavalent vaccine inject on the thigh? __ yes 🡪 3.33

They sometimes give it the same time they can put the polio vaccine in the mouth. __ no 🡪 3.34

__ don’t know 🡪 3.34

**LAST MILE SURVEY – Child Questionnaire** **(page 3)**

1. How many times they ever give (NAME) pentavalent vaccine _____________ (number)

injection on the thigh like that? __ don’t know

1. They ever give (NAME) an injection in the thigh to prevent pneumonia? ___ yes 🡪 3.35

___ no 🡪 3.36

___ don’t know 🡪 3.36

1. How many times did (NAME) get the injection in the thigh to prevent pneumonia? _____________ (number)

__ don’t know

1. They ever give (NAME) a rotavirus vaccination, that is, a liquid in the ___ yes 🡪 3.37

mouth to prevent diarrhea? ___ no 🡪 3.38

___ don’t know 🡪 3.38

1. How many times did (NAME) receive the rotavirus vaccine? _____________ (number)

__ don’t know

1. They ever give (NAME) vaccine for Measles sickness? That the vaccine ___ yes

Injection they can give the children in the arm when they are 9 months or more. ___ no

___ don’t know

**LAST MILE SURVEY – General Health Questionnaire** **(page 1)**

1. In the last four weeks, how many times have you gCHV? ________

or anyone in this household visited or used a… [READ ALL] FHW? _________

Hospital/clinic? ______

Drugstore? _______

Tablet man/black bagger? ______

Country doctor? ________

Other (__________)? _________

_____________________________________________________________________________________________________________________________________________________

1. Where you go to get medical advice or treatment? [MORE THAN ONE] __ to see doctor/nurse at clinic/hospital

__to the drugstore __to tablet man/black bagger __to see gCHV __to see FHW __ to country doctor __other: ______

_____________________________________________________________________________________________________________________________________________________

1. In the last year, you or anyone in your family ever need medical ___ yes 🡪 4.4

advice or treatment but not able to get it? ___ no

_____________________________________________________________________________________________________________________________________________________

1. What things stopping you from getting medical treatment? Any other thing? [MORE THAN ONE]

__ no transport money __ no money for treatment __ afraid to go to provider __no place to stay near provider

__ distance to provider __ no medicine available __ no permission to go __no one to take care of family

__ provider not available __ Ebola outbreak __ other: ___________________

**EBOLA MODULE**

1. In your own thinking, what are the signs of someone who can have Ebola? [DO NOT READ, MORE THAN ONE]

| Fever | Sore throat | Other: __________ |
| --- | --- | --- |
| Muscle pains | Running stomach |  |
| Vomiting | Bleeding from eyes, mouth, nose |  |
| No response | Ebola is not real |  |

1. Can people can get Ebola by touching an Ebola patient? __ yes __ no __ don’t know
2. Can people get Ebola from the air? __ yes __ no __ don’t know
3. Can people get Ebola from touching or washing a dead body? __ yes __ no __ don’t know
4. Can people get Ebola from touching the vomit of an Ebola patient? __ yes __ no __ don’t know
5. Can people get Ebola because of witchcraft or something like that? __ yes __ no __ don’t know
6. Can people get Ebola by going to the hospital/clinic? __ yes __ no __ don’t know
7. If it looking like you or someone in your family could have Ebola, would ___yes 🡪 4.13 you try to get medical advice or treatment? ____ no
8. Where would you go **FIRST**? [ONLY ONE]__ to see family member, friend, or neighbor __ to see FHW __ to see gCHV __ to see doctor or nurse at the clinic __to the ETU __ to the tablet man/black bagger __ to country doctor __ to other: ____
