# Supplementary material for: Facility-Based Delivery during the Ebola Virus Disease Epidemic in Rural Liberia: Analysis from a Cross-Sectional, Population-Based Household Survey
Source: PLoS Med. 2016 Aug 2;13(8):e1002096. doi: 10.1371/journal.pmed.1002096 (PMC4970816; doi:10.1371/journal.pmed.1002096)
Supplement: S1 Table — (DOCX) [file pmed.1002096.s009.docx]

| **Supplemental Table 1.** Analysis restricted only to those respondents who reported a belief that clinics did or may pose an Ebola transmission risk. | | | | | | | | |
| --- | --- | --- | --- | --- | --- | --- | --- | --- |
|  | **Unadjusted Model** | | **Multivariable Model 1** | | **Multivariable Model 2** | | **Multivariable Model 3** | |
|  | OR (95% CI) | p | AOR (95% CI) | p | AOR (95% CI) | p | AOR (95% CI) | p |
|  |  |  |  |  |  |  |  |  |
| Ebola period | 0.58 (0.37-0.91) | 0.019 | 0.59 (0.36-0.97) | 0.038 | 0.58 (0.35-0.94) | 0.029 | 0.58 (0.36-0.94) | 0.027 |
| Household wealth |  |  | 1.38 (1.07-1.78) | 0.015 | 1.22 (0.94-1.58) | 0.125 | 1.20 (0.94-1.52) | 0.141 |
| Maternal education |  |  |  |  |  |  |  |  |
| None |  |  | Ref. | Ref. | Ref. | Ref. | Ref. | Ref. |
| Primary only |  |  | 1.23 (0.74-2.05) | 0.426 | 1.15 (0.69-1.91) | 0.598 | 1.13 (0.65-1.94) | 0.666 |
| Secondary or higher |  |  | 1.51 (0.78-2.91) | 0.216 | 1.48 (0.75-2.92) | 0.256 | 1.44 (0.72-2.89) | 0.300 |
| Bassa language speaker |  |  |  |  | 0.96 (0.58-1.60) | 0.879 | 0.94 (0.58-1.52) | 0.790 |
| Distance from health facility |  |  |  |  |  |  |  |  |
| Per km, up to 10km |  |  |  |  | 0.89 (0.82-0.98) | 0.016 | 0.89 (0.82-0.98) | 0.017 |
| Per km, 10 to 21km |  |  |  |  | 1.00 (0.92-1.09) | 0.953 | 0.99 (0.91-1.08) | 0.865 |
| Per km, 21km and over |  |  |  |  | 0.91 (0.81-1.01) | 0.089 | 0.92 (0.82-1.03) | 0.134 |
| Maternal age at birth |  |  |  |  |  |  |  |  |
| First quartile |  |  |  |  |  |  | Ref. | Ref. |
| Second quartile |  |  |  |  |  |  | 0.47 (0.24-0.92) | 0.029 |
| Third quartile |  |  |  |  |  |  | 0.68 (0.36-1.28) | 0.231 |
| Fourth quartile |  |  |  |  |  |  | 0.75 (0.39-1.47) | 0.399 |
| Mother is married |  |  |  |  |  |  | 0.79 (0.43-1.43) | 0.428 |
| Birth order |  |  |  |  |  |  |  |  |
| 1^st^ |  |  |  |  |  |  | Ref. | Ref. |
| 2^nd^ or 3^rd^ |  |  |  |  |  |  | 0.83 (0.52-1.33) | 0.441 |
| 4^th^ or higher |  |  |  |  |  |  | 0.92 (0.54-1.56) | 0.759 |
| Rainy season birth |  |  |  |  |  |  | 1.08 (0.70-1.67) | 0.727 |
|  | | | | | | | | |
