# Supplementary material for: Facility-Based Delivery during the Ebola Virus Disease Epidemic in Rural Liberia: Analysis from a Cross-Sectional, Population-Based Household Survey
Source: PLoS Med. 2016 Aug 2;13(8):e1002096. doi: 10.1371/journal.pmed.1002096 (PMC4970816; doi:10.1371/journal.pmed.1002096)
Supplement: S2 Table — (DOCX) [file pmed.1002096.s010.docx]

| **Supplemental Table 2.** Analysis restricted only to those respondents who reported a belief that clinics did not pose an Ebola transmission risk. | | | | | | | | |
| --- | --- | --- | --- | --- | --- | --- | --- | --- |
|  | **Unadjusted Model** | | **Multivariable Model 1** | | **Multivariable Model 2** | | **Multivariable Model 3** | |
|  | OR (95% CI) | p | AOR (95% CI) | p | AOR (95% CI) | p | AOR (95% CI) | p |
|  |  |  |  |  |  |  |  |  |
| Ebola period | 0.81 (0.52-0.1.26) | 0.346 | 0.90 (0.59-1.37) | 0.612 | 0.93 (0.60-1.45) | 0.761 | 0.83 (0.53-1.30) | 0.405 |
| Household wealth |  |  | 2.16 (1.20-3.87) | 0.010 | 1.21 (0.63-2.30) | 0.565 | 1.18 (0.63-2.20) | 0.594 |
| Maternal education |  |  |  |  |  |  |  |  |
| None |  |  | Ref. | Ref. | Ref. | Ref. | Ref. | Ref. |
| Primary only |  |  | 1.00 (0.57-1.76) | 0.991 | 0.93 (0.54-1.59) | 0.776 | 0.86 (0.48-1.57) | 0.628 |
| Secondary or higher |  |  | 1.98 (0.76-5.12) | 0.158 | 3.30 (1.22-8.95) | 0.020 | 3.31 (1.11-9.85) | 0.032 |
| Bassa language speaker |  |  |  |  | 0.47 (0.26-0.83) | 0.011 | 0.39 (0.20-0.74) | 0.005 |
| Distance from health facility |  |  |  |  |  |  |  |  |
| Per km, up to 10km |  |  |  |  | 0.79 (0.64-0.96) | 0.020 | 0.77 (0.63-0.94) | 0.011 |
| Per km, 10 to 21km |  |  |  |  | 1.01 (0.90-1.13) | 0.861 | 1.02 (0.92-1.14) | 0.663 |
| Per km, 21km and over |  |  |  |  | 0.88 (0.79-0.98) | 0.017 | 0.86 (0.77-0.95) | 0.004 |
| Maternal age at birth |  |  |  |  |  |  |  |  |
| First quartile |  |  |  |  |  |  | Ref. | Ref. |
| Second quartile |  |  |  |  |  |  | 1.37 (0.59-3.17) | 0.463 |
| Third quartile |  |  |  |  |  |  | 0.86 (0.46-1.61) | 0.626 |
| Fourth quartile |  |  |  |  |  |  | 0.68 (0.36-1.27) | 0.219 |
| Mother is married |  |  |  |  |  |  | 1.79 (0.76-4.20) | 0.178 |
| Birth order |  |  |  |  |  |  |  |  |
| 1^st^ |  |  |  |  |  |  | Ref. | Ref. |
| 2^nd^ or 3^rd^ |  |  |  |  |  |  | 1.05 (0.54-2.06) | 0.884 |
| 4^th^ or higher |  |  |  |  |  |  | 1.97 (0.96-4.04) | 0.062 |
| Rainy season birth |  |  |  |  |  |  | 0.66 (0.40-1.10) | 0.112 |
|  | | | | | | | | |
