# Supplementary material for: Facility-Based Delivery during the Ebola Virus Disease Epidemic in Rural Liberia: Analysis from a Cross-Sectional, Population-Based Household Survey
Source: PLoS Med. 2016 Aug 2;13(8):e1002096. doi: 10.1371/journal.pmed.1002096 (PMC4970816; doi:10.1371/journal.pmed.1002096)
Supplement: S3 Table — (DOC) [file pmed.1002096.s011.doc]

| **Supplemental Table 3.** Sensitivity Analysis: Restricted to only households who lived in current village at the time of the delivery. N=818 | | | | | | | | |
| --- | --- | --- | --- | --- | --- | --- | --- | --- |
|  | **Unadjusted Model** | | **Multivariable Model 1** | | **Multivariable Model 2** | | **Multivariable Model 3** | |
|  | OR (95% CI) | p | AOR (95% CI) | p | AOR (95% CI) | p | AOR (95% CI) | p |
|  |  |  |  |  |  |  |  |  |
| Ebola period | 0.70 (0.52-0.95) | 0.024 | 0.72 (0.52-1.01) | 0.056 | 0.73 (0.52-1.02) | 0.065 | 0.73 (0.52-1.02) | 0.067 |
| Household wealth |  |  | 1.72 (1.28-2.30) | 0.001 | 1.27 (0.98-1.65) | 0.068 | 1.29 (0.99-1.68) | 0.057 |
| Maternal education |  |  |  |  |  |  |  |  |
| None |  |  | Ref. | Ref. | Ref. | Ref. | Ref. | Ref. |
| Primary only |  |  | 1.15 (0.78-1.70) | 0.486 | 1.05 (0.72-1.52) | 0.809 | 1.00 (0.67-1.49) | 0.995 |
| Secondary or higher |  |  | 1.19 (0.63-2.25) | 0.589 | 1.25 (0.63-2.49) | 0.514 | 1.18 (0.57-2.45) | 0.649 |
| Bassa language speaker |  |  |  |  | 0.81 (0.52-1.28) | 0.370 | 0.79 (0.50-1.27) | 0.325 |
| Distance from health facility |  |  |  |  |  |  |  |  |
| Per km, up to 10km |  |  |  |  | 0.85 (0.78-0.93) | <0.001 | 0.85 (0.78-0.93) | <0.001 |
| Per km, 10 to 21km |  |  |  |  | 0.99 (0.91-1.07) | 0.718 | 0.99 (0.91-1.07) | 0.713 |
| Per km, 21km and over |  |  |  |  | 0.91 (0.80-1.03) | 0.123 | 0.91 (0.80-1.04) | 0.154 |
| Maternal age at birth |  |  |  |  |  |  |  |  |
| First quartile |  |  |  |  |  |  | Ref. | Ref. |
| Second quartile |  |  |  |  |  |  | 0.77 (0.48-1.23) | 0.264 |
| Third quartile |  |  |  |  |  |  | 0.68 (0.46-1.03) | 0.066 |
| Fourth quartile |  |  |  |  |  |  | 0.73 (0.45-1.16) | 0.181 |
| Mother is married |  |  |  |  |  |  | 1.04 (0.61-1.76) | 0.880 |
| Birth order |  |  |  |  |  |  |  |  |
| 1^st^ |  |  |  |  |  |  | Ref. | Ref. |
| 2^nd^ or 3^rd^ |  |  |  |  |  |  | 0.78 (0.54-1.13) | 0.193 |
| 4^th^ or higher |  |  |  |  |  |  | 1.08 (0.74-1.58) | 0.693 |
| Rainy season birth |  |  |  |  |  |  | 0.84 (0.60-1.17) | 0.289 |
|  | | | | | | | | |
