# Supplementary material for: Facility-Based Delivery during the Ebola Virus Disease Epidemic in Rural Liberia: Analysis from a Cross-Sectional, Population-Based Household Survey
Source: PLoS Med. 2016 Aug 2;13(8):e1002096. doi: 10.1371/journal.pmed.1002096 (PMC4970816; doi:10.1371/journal.pmed.1002096)
Supplement: S4 Table — (DOC) [file pmed.1002096.s012.doc]

| **Supplemental Table 4.** Sensitivity Analysis: Excludes births within two weeks before the survey began. N=884 | | | | | | | | |
| --- | --- | --- | --- | --- | --- | --- | --- | --- |
|  | **Unadjusted Model** | | **Multivariable Model 1** | | **Multivariable Model 2** | | **Multivariable Model 3** | |
|  | OR (95% CI) | p | AOR (95% CI) | p | AOR (95% CI) | p | AOR (95% CI) | p |
|  |  |  |  |  |  |  |  |  |
| Ebola period | 0.65 (0.48-0.89) | 0.009 | 0.69 (0.49-0.97) | 0.032 | 0.68 (0.49-0.96) | 0.028 | 0.68 (0.49-0.96) | 0.028 |
| Household wealth |  |  | 1.67 (1.28-2.16) | <0.001 | 1.23 (0.97-1.56) | 0.084 | 1.24 (0.98-1.57) | 0.075 |
| Maternal education |  |  |  |  |  |  |  |  |
| None |  |  | Ref. | Ref. | Ref. | Ref. | Ref. | Ref. |
| Primary only |  |  | 1.18 (0.80-1.75) | 0.396 | 1.08 (0.75-1.57) | 0.670 | 1.05 (0.71-1.55) | 0.794 |
| Secondary or higher |  |  | 1.44 (0.79-2.63) | 0.229 | 1.52 (0.82-2.81) | 0.179 | 1.52 (0.79-2.92) | 0.204 |
| Bassa language speaker |  |  |  |  | 0.73 (0.48-1.12) | 0.143 | 0.72 (0.46-1.12) | 0.141 |
| Distance from health facility |  |  |  |  |  |  |  |  |
| Per km, up to 10km |  |  |  |  | 0.85 (0.78-0.92) | <0.001 | 0.85 (0.78-0.92) | <0.001 |
| Per km, 10 to 21km |  |  |  |  | 1.00 (0.93-1.08) | 0.985 | 1.00 (0.93-1.08) | 0.977 |
| Per km, 21km and over |  |  |  |  | 0.91 (0.83-1.00) | 0.050 | 0.91 (0.83-1.01) | 0.064 |
| Maternal age at birth |  |  |  |  |  |  |  |  |
| First quartile |  |  |  |  |  |  | Ref. | Ref. |
| Second quartile |  |  |  |  |  |  | 0.73 (0.45-1.17) | 0.186 |
| Third quartile |  |  |  |  |  |  | 0.73 (0.49-1.10) | 0.129 |
| Fourth quartile |  |  |  |  |  |  | 0.76 (0.48-1.20) | 0.231 |
| Mother is married |  |  |  |  |  |  | 1.05 (0.64-1.72) | 0.845 |
| Birth order |  |  |  |  |  |  |  |  |
| 1^st^ |  |  |  |  |  |  | Ref. | Ref. |
| 2^nd^ or 3^rd^ |  |  |  |  |  |  | 0.88 (0.61-1.26) | 0.471 |
| 4^th^ or higher |  |  |  |  |  |  | 1.16 (0.78-1.71) | 0.459 |
| Rainy season birth |  |  |  |  |  |  | 0.87 (0.63-1.20) | 0.397 |
|  | | | | | | | | |
