# Supplementary material for: Facility-Based Delivery during the Ebola Virus Disease Epidemic in Rural Liberia: Analysis from a Cross-Sectional, Population-Based Household Survey
Source: PLoS Med. 2016 Aug 2;13(8):e1002096. doi: 10.1371/journal.pmed.1002096 (PMC4970816; doi:10.1371/journal.pmed.1002096)
Supplement: S5 Table — (DOC) [file pmed.1002096.s013.doc]

| **Supplemental Table 5.** Sensitivity Analysis: Excludes women over age 45 at time of birth to avoid possible bias from attrition. N=895 | | | | | | | | |
| --- | --- | --- | --- | --- | --- | --- | --- | --- |
|  | **Unadjusted Model** | | **Multivariable Model 1** | | **Multivariable Model 2** | | **Multivariable Model 3** | |
|  | OR (95% CI) | p | AOR (95% CI) | p | AOR (95% CI) | p | AOR (95% CI) | p |
|  |  |  |  |  |  |  |  |  |
| Ebola period | 0.65 (0.48-0.90) | 0.009 | 0.69 (0.49-0.97) | 0.034 | 0.69 (0.49-0.97) | 0.033 | 0.69 (0.49-0.97) | 0.031 |
| Household wealth |  |  | 1.67 (1.29-2.17) | <0.001 | 1.25 (0.99-1.58) | 0.065 | 1.26 (0.99-1.60) | 0.058 |
| Maternal education |  |  |  |  |  |  |  |  |
| None |  |  | Ref. | Ref. | Ref. | Ref. | Ref. | Ref. |
| Primary only |  |  | 1.18 (0.80-1.73) | 0.401 | 1.09 (0.76-1.57) | 0.640 | 1.05 (0.71-1.54) | 0.812 |
| Secondary or higher |  |  | 1.39 (0.77-2.50) | 0.272 | 1.49 (0.81-2.74) | 0.193 | 1.47 (0.77-2.83) | 0.239 |
| Bassa language speaker |  |  |  |  | 0.77 (0.50-1.18) | 0.221 | 0.76 (0.49-1.18) | 0.211 |
| Distance from health facility |  |  |  |  |  |  |  |  |
| Per km, up to 10km |  |  |  |  | 0.85 (0.78-0.92) | <0.001 | 0.85 (0.78-0.92) | <0.001 |
| Per km, 10 to 21km |  |  |  |  | 1.00 (0.93-1.08) | 0.991 | 1.00 (0.93-1.08) | 0.963 |
| Per km, 21km and over |  |  |  |  | 0.91 (0.83-1.00) | 0.057 | 0.91 (0.83-1.01) | 0.075 |
| Maternal age at birth |  |  |  |  |  |  |  |  |
| First quartile |  |  |  |  |  |  | Ref. | Ref. |
| Second quartile |  |  |  |  |  |  | 0.73 (0.46-1.18) | 0.194 |
| Third quartile |  |  |  |  |  |  | 0.71 (0.48-1.07) | 0.098 |
| Fourth quartile |  |  |  |  |  |  | 0.74 (0.46-1.16) | 0.179 |
| Mother is married |  |  |  |  |  |  | 1.03 (0.63-1.68) | 0.904 |
| Birth order |  |  |  |  |  |  |  |  |
| 1^st^ |  |  |  |  |  |  | Ref. | Ref. |
| 2^nd^ or 3^rd^ |  |  |  |  |  |  | 0.89 (0.62-1.29) | 0.534 |
| 4^th^ or higher |  |  |  |  |  |  | 1.16 (0.78-1.71) | 0.460 |
| Rainy season birth |  |  |  |  |  |  | 0.87 (0.63-1.20) | 0.397 |
|  | | | | | | | | |
