# Supplementary material for: Facility-Based Delivery during the Ebola Virus Disease Epidemic in Rural Liberia: Analysis from a Cross-Sectional, Population-Based Household Survey
Source: PLoS Med. 2016 Aug 2;13(8):e1002096. doi: 10.1371/journal.pmed.1002096 (PMC4970816; doi:10.1371/journal.pmed.1002096)
Supplement: S6 Table — (DOC) [file pmed.1002096.s014.doc]

| **Supplemental Table 6.** Sensitivity Analysis: Excludes births within two weeks of the survey, women over age 45 at the time of delivery, and women who lived in a different village at the time of delivery. N=801 | | | | | | | | |
| --- | --- | --- | --- | --- | --- | --- | --- | --- |
|  | **Unadjusted Model** | | **Multivariable Model 1** | | **Multivariable Model 2** | | **Multivariable Model 3** | |
|  | OR (95% CI) | p | AOR (95% CI) | p | AOR (95% CI) | p | AOR (95% CI) | p |
|  |  |  |  |  |  |  |  |  |
| Ebola period | 0.69 (0.50-0.94) | 0.020 | 0.70 (0.50-0.99) | 0.044 | 0.71 (0.51-1.00) | 0.051 | 0.72 (0.52-1.02) | 0.061 |
| Household wealth |  |  | 1.71 (1.28-2.28) | <0.001 | 1.26 (0.97-1.63) | 0.077 | 1.28 (0.99-1.66) | 0.064 |
| Maternal education |  |  |  |  |  |  |  |  |
| None |  |  | Ref. | Ref. | Ref. | Ref. | Ref. | Ref. |
| Primary only |  |  | 1.13 (0.76-1.69) | 0.532 | 1.03 (0.71-1.51) | 0.870 | 0.99 (0.66-1.48) | 0.962 |
| Secondary or higher |  |  | 1.14 (0.59-2.18) | 0.697 | 1.19 (0.59-2.39) | 0.627 | 1.12 (0.53-2.37) | 0.766 |
| Bassa language speaker |  |  |  |  | 0.77 (0.49-1.22) | 0.258 | 0.75 (0.47-1.20) | 0.229 |
| Distance from health facility |  |  |  |  |  |  |  |  |
| Per km, up to 10km |  |  |  |  | 0.85 (0.78-0.93) | <0.001 | 0.85 (0.78-0.93) | <0.001 |
| Per km, 10 to 21km |  |  |  |  | 0.98 (0.91-1.07) | 0.706 | 0.98 (0.91-1.07) | 0.712 |
| Per km, 21km and over |  |  |  |  | 0.91 (0.80-1.03) | 0.121 | 0.91 (0.80-1.04) | 0.150 |
| Maternal age at birth |  |  |  |  |  |  |  |  |
| First quartile |  |  |  |  |  |  | Ref. | Ref. |
| Second quartile |  |  |  |  |  |  | 0.76 (0.47-1.22) | 0.256 |
| Third quartile |  |  |  |  |  |  | 0.70 (0.46-1.05) | 0.086 |
| Fourth quartile |  |  |  |  |  |  | 0.72 (0.45-1.16) | 0.170 |
| Mother is married |  |  |  |  |  |  | 1.05 (0.62-1.78) | 0.865 |
| Birth order |  |  |  |  |  |  |  |  |
| 1^st^ |  |  |  |  |  |  | Ref. | Ref. |
| 2^nd^ or 3^rd^ |  |  |  |  |  |  | 0.78 (0.54-1.13) | 0.181 |
| 4^th^ or higher |  |  |  |  |  |  | 1.07 (0.73-1.57) | 0.709 |
| Rainy season birth |  |  |  |  |  |  | 0.85 (0.60-1.19) | 0.328 |
|  | | | | | | | | |
