# Supplementary material for: Facility-Based Delivery during the Ebola Virus Disease Epidemic in Rural Liberia: Analysis from a Cross-Sectional, Population-Based Household Survey
Source: PLoS Med. 2016 Aug 2;13(8):e1002096. doi: 10.1371/journal.pmed.1002096 (PMC4970816; doi:10.1371/journal.pmed.1002096)
Supplement: S7 Table — (DOC) [file pmed.1002096.s015.doc]

| **Supplemental Table 7.** Sensitivity Analysis: Sensitivity Analysis: Includes observations from 2012 to 2015. N=722 | | | | | | | | |
| --- | --- | --- | --- | --- | --- | --- | --- | --- |
|  | **Unadjusted Model** | | **Multivariable Model 1** | | **Multivariable Model 2** | | **Multivariable Model 3** | |
|  | OR (95% CI) | p | AOR (95% CI) | p | AOR (95% CI) | p | AOR (95% CI) | p |
|  |  |  |  |  |  |  |  |  |
| Ebola period | 0.66 (0.48-0.91) | 0.011 | 0.70 (0.50-0.98) | 0.037 | 0.69 (0.49-0.95) | 0.028 | 0.68 (0.49-0.95) | 0.026 |
| Household wealth |  |  | 1.72 (1.27-2.32) | 0.001 | 1.30 (0.98-1.72) | 0.071 | 1.30 (0.98-1.72) | 0.066 |
| Maternal education |  |  |  |  |  |  |  |  |
| None |  |  | Ref. | Ref. | Ref. | Ref. | Ref. | Ref. |
| Primary only |  |  | 1.10 (0.74-1.65) | 0.635 | 1.02 (0.69-1.51) | 0.912 | 0.99 (0.67-1.46) | 0.943 |
| Secondary or higher |  |  | 1.16 (0.64-2.09) | 0.615 | 1.27 (0.68-2.37) | 0.441 | 1.26 (0.65-2.45) | 0.488 |
| Bassa language speaker |  |  |  |  | 0.82 (0.51-1.31) | 0.396 | 0.80 (0.49-1.31) | 0.379 |
| Distance from health facility |  |  |  |  |  |  |  |  |
| Per km, up to 10km |  |  |  |  | 0.83 (0.74-0.93) | 0.001 | 0.83 (0.74-0.92) | 0.001 |
| Per km, 10 to 21km |  |  |  |  | 1.01 (0.92-1.10) | 0.887 | 1.01 (0.92-1.10) | 0.870 |
| Per km, 21km and over |  |  |  |  | 0.94 (0.84-1.05) | 0.253 | 0.94 (0.83-1.06) | 0.318 |
| Maternal age at birth |  |  |  |  |  |  |  |  |
| First quartile |  |  |  |  |  |  | Ref. | Ref. |
| Second quartile |  |  |  |  |  |  | 0.89 (0.54-1.47) | 0.639 |
| Third quartile |  |  |  |  |  |  | 0.74 (0.47-1.16) | 0.181 |
| Fourth quartile |  |  |  |  |  |  | 0.83 (0.50-1.41) | 0.492 |
| Mother is married |  |  |  |  |  |  | 0.95 (0.53-1.72) | 0.873 |
| Birth order |  |  |  |  |  |  |  |  |
| 1^st^ |  |  |  |  |  |  | Ref. | Ref. |
| 2^nd^ or 3^rd^ |  |  |  |  |  |  | 0.81 (0.55-1.21) | 0.305 |
| 4^th^ or higher |  |  |  |  |  |  | 1.20 (0.76-1.88) | 0.426 |
| Rainy season birth |  |  |  |  |  |  | 0.90 (0.64-1.26) | 0.529 |
|  | | | | | | | | |
