# Supplementary material for: Facility-Based Delivery during the Ebola Virus Disease Epidemic in Rural Liberia: Analysis from a Cross-Sectional, Population-Based Household Survey
Source: PLoS Med. 2016 Aug 2;13(8):e1002096. doi: 10.1371/journal.pmed.1002096 (PMC4970816; doi:10.1371/journal.pmed.1002096)
Supplement: S8 Table — (DOC) [file pmed.1002096.s016.doc]

| **Supplemental Table 8.** Sensitivity Analysis: Includes observations from 2013 to 2015. N=537 | | | | | | | | |
| --- | --- | --- | --- | --- | --- | --- | --- | --- |
|  | **Unadjusted Model** | | **Multivariable Model 1** | | **Multivariable Model 2** | | **Multivariable Model 3** | |
|  | OR (95% CI) | p | AOR (95% CI) | p | AOR (95% CI) | p | AOR (95% CI) | p |
|  |  |  |  |  |  |  |  |  |
| Ebola period | 0.68 (0.49-0.95) | 0.025 | 0.72 (0.51-1.01) | 0.060 | 0.72 (0.51-1.02) | 0.067 | 0.70 (0.50-1.00) | 0.048 |
| Household wealth |  |  | 1.67 (1.17-2.39) | 0.006 | 1.32 (0.94-1.85) | 0.110 | 1.33 (0.95-1.85) | 0.094 |
| Maternal education |  |  |  |  |  |  |  |  |
| None |  |  | Ref. | Ref. | Ref. | Ref. | Ref. | Ref. |
| Primary only |  |  | 1.05 (0.68-1.61) | 0.835 | 1.00 (0.67-1.50) | 0.994 | 0.98 (0.65-1.49) | 0.933 |
| Secondary or higher |  |  | 1.05 (0.54-2.04) | 0.883 | 1.28 (0.61-2.68) | 0.514 | 1.34 (0.62-2.89) | 0.452 |
| Bassa language speaker |  |  |  |  | 1.04 (0.64-1.71) | 0.869 | 1.04 (0.61-1.80) | 0.873 |
| Distance from health facility |  |  |  |  |  |  |  |  |
| Per km, up to 10km |  |  |  |  | 0.83 (0.72-0.96) | 0.011 | 0.83 (0.72-0.96) | 0.010 |
| Per km, 10 to 21km |  |  |  |  | 0.98 (0.90-1.07) | 0.681 | 0.98 (0.90-1.07) | 0.702 |
| Per km, 21km and over |  |  |  |  | 0.96 (0.87-1.06) | 0.385 | 0.96 (0.86-1.07) | 0.435 |
| Maternal age at birth |  |  |  |  |  |  |  |  |
| First quartile |  |  |  |  |  |  | Ref. | Ref. |
| Second quartile |  |  |  |  |  |  | 0.85 (0.46-1.59) | 0.614 |
| Third quartile |  |  |  |  |  |  | 0.70 (0.42-1.19) | 0.184 |
| Fourth quartile |  |  |  |  |  |  | 0.91 (0.49-1.71) | 0.770 |
| Mother is married |  |  |  |  |  |  | 0.95 (0.49-1.84) | 0.868 |
| Birth order |  |  |  |  |  |  |  |  |
| 1^st^ |  |  |  |  |  |  | Ref. | Ref. |
| 2^nd^ or 3^rd^ |  |  |  |  |  |  | 0.99 (0.63-1.57) | 0.966 |
| 4^th^ or higher |  |  |  |  |  |  | 1.27 (0.78-2.08) | 0.327 |
| Rainy season birth |  |  |  |  |  |  | 0.83 (0.58-1.20) | 0.326 |
|  | | | | | | | | |
