# Supplementary material for: Facility-Based Delivery during the Ebola Virus Disease Epidemic in Rural Liberia: Analysis from a Cross-Sectional, Population-Based Household Survey
Source: PLoS Med. 2016 Aug 2;13(8):e1002096. doi: 10.1371/journal.pmed.1002096 (PMC4970816; doi:10.1371/journal.pmed.1002096)
Supplement: S9 Table — (DOC) [file pmed.1002096.s017.doc]

| **Supplemental Table 9.** Sensitivity Analysis: Includes observations from 2010 to 2015. N=1072 | | | | | | | | |
| --- | --- | --- | --- | --- | --- | --- | --- | --- |
|  | **Unadjusted Model** | | **Multivariable Model 1** | | **Multivariable Model 2** | | **Multivariable Model 3** | |
|  | OR (95% CI) | p | AOR (95% CI) | p | AOR (95% CI) | p | AOR (95% CI) | p |
|  |  |  |  |  |  |  |  |  |
| Ebola period | 0.70 (0.52-0.96) | 0.028 | 0.75 (0.54-1.04) | 0.088 | 0.76 (0.55-1.05) | 0.094 | 0.76 (0.55-1.05) | 0.099 |
| Household wealth |  |  | 1.58 (1.25-1.99) | <0.001 | 1.21 (0.98-1.50) | 0.079 | 1.21 (0.97-1.51) | 0.084 |
| Maternal education |  |  |  |  |  |  |  |  |
| None |  |  | Ref. | Ref. | Ref. | Ref. | Ref. | Ref. |
| Primary only |  |  | 1.12 (0.76-1.64) | 0.555 | 1.04 (0.72-1.49) | 0.845 | 1.00 (0.68-1.47) | 0.996 |
| Secondary or higher |  |  | 1.22 (0.62-2.39) | 0.559 | 1.29 (0.61-2.74) | 0.494 | 1.27 (0.59-2.73) | 0.533 |
| Bassa language speaker |  |  |  |  | 0.85 (0.59-1.23) | 0.391 | 0.85 (0.57-1.25) | 0.395 |
| Distance from health facility |  |  |  |  |  |  |  |  |
| Per km, up to 10km |  |  |  |  | 0.86 (0.80-0.93) | <0.001 | 0.86 (0.80-0.93) | <0.001 |
| Per km, 10 to 21km |  |  |  |  | 1.00 (0.93-1.07) | 0.936 | 1.00 (0.93-1.07) | 0.940 |
| Per km, 21km and over |  |  |  |  | 0.93 (0.85-1.01) | 0.092 | 0.93 (0.86-1.02) | 0.117 |
| Maternal age at birth |  |  |  |  |  |  |  |  |
| First quartile |  |  |  |  |  |  | Ref. | Ref. |
| Second quartile |  |  |  |  |  |  | 0.82 (0.51-1.32) | 0.404 |
| Third quartile |  |  |  |  |  |  | 0.74 (0.50-1.09) | 0.127 |
| Fourth quartile |  |  |  |  |  |  | 0.82 (0.54-1.24) | 0.349 |
| Mother is married |  |  |  |  |  |  | 1.02 (0.61-1.71) | 0.947 |
| Birth order |  |  |  |  |  |  |  |  |
| 1^st^ |  |  |  |  |  |  | Ref. | Ref. |
| 2^nd^ or 3^rd^ |  |  |  |  |  |  | 0.79 (0.54-1.16) | 0.227 |
| 4^th^ or higher |  |  |  |  |  |  | 1.03 (0.72-1.50) | 0.854 |
| Rainy season birth |  |  |  |  |  |  | 0.84 (0.63-1.11) | 0.214 |
|  | | | | | | | | |
