# Supplementary material for: Facility-Based Delivery during the Ebola Virus Disease Epidemic in Rural Liberia: Analysis from a Cross-Sectional, Population-Based Household Survey
Source: PLoS Med. 2016 Aug 2;13(8):e1002096. doi: 10.1371/journal.pmed.1002096 (PMC4970816; doi:10.1371/journal.pmed.1002096)
Supplement: S10 Table — (DOC) [file pmed.1002096.s018.doc]

| **Supplemental Table 10.** Sensitivity Analysis: Jackknifed standard errors instead of Taylor linearization. N=898 | | | | | | | | |
| --- | --- | --- | --- | --- | --- | --- | --- | --- |
|  | **Unadjusted Model** | | **Multivariable Model 1** | | **Multivariable Model 2** | | **Multivariable Model 3** | |
|  | OR (95% CI) | p | AOR (95% CI) | p | AOR (95% CI) | p | AOR (95% CI) | p |
|  |  |  |  |  |  |  |  |  |
| Ebola period | 0.66 (0.49-0.88) | 0.005 | 0.70 (0.51-0.95) | 0.025 | 0.69 (0.51-0.95) | 0.024 | 0.69 (0.51-0.95) | 0.023 |
| Household wealth |  |  | 1.67 (1.31-2.13) | <0.001 | 1.25 (1.00-1.55) | 0.050 | 1.26 (1.00-1.57) | 0.048 |
| Maternal education |  |  |  |  |  |  |  |  |
| None |  |  | Ref. | Ref. | Ref. | Ref. | Ref. | Ref. |
| Primary only |  |  | 1.19 (0.82-1.72) | 0.359 | 1.09 (0.77-1.55) | 0.611 | 1.05 (0.73-1.53) | 0.776 |
| Secondary or higher |  |  | 1.44 (0.82-2.50) | 0.197 | 1.54 (0.87-2.73) | 0.136 | 1.53 (0.82-2.85) | 0.176 |
| Bassa language speaker |  |  |  |  | 0.77 (0.50-1.19) | 0.230 | 0.76 (0.48-1.20) | 0.229 |
| Distance from health facility |  |  |  |  |  |  |  |  |
| Per km, up to 10km |  |  |  |  | 0.85 (0.78-0.92) | <0.001 | 0.85 (0.78-0.92) | <0.001 |
| Per km, 10 to 21km |  |  |  |  | 1.00 (0.92-1.09) | 0.994 | 1.00 (0.92-1.09) | 0.969 |
| Per km, 21km and over |  |  |  |  | 0.91 (0.75-1.10) | 0.315 | 0.91 (0.74-1.12) | 0.377 |
| Maternal age at birth |  |  |  |  |  |  |  |  |
| First quartile |  |  |  |  |  |  | Ref. | Ref. |
| Second quartile |  |  |  |  |  |  | 0.73 (0.47-1.15) | 0.170 |
| Third quartile |  |  |  |  |  |  | 0.71 (0.48-1.05) | 0.086 |
| Fourth quartile |  |  |  |  |  |  | 0.75 (0.48-1.16) | 0.190 |
| Mother is married |  |  |  |  |  |  | 1.04 (0.63-1.70) | 0.883 |
| Birth order |  |  |  |  |  |  |  |  |
| 1^st^ |  |  |  |  |  |  | Ref. | Ref. |
| 2^nd^ or 3^rd^ |  |  |  |  |  |  | 0.89 (0.63-1.25) | 0.492 |
| 4^th^ or higher |  |  |  |  |  |  | 1.16 (0.80-1.68) | 0.431 |
| Rainy season birth |  |  |  |  |  |  | 0.87 (0.64-1.17) | 0.340 |
|  | | | | | | | | |
