# Supplementary material for: Facility-Based Delivery during the Ebola Virus Disease Epidemic in Rural Liberia: Analysis from a Cross-Sectional, Population-Based Household Survey
Source: PLoS Med. 2016 Aug 2;13(8):e1002096. doi: 10.1371/journal.pmed.1002096 (PMC4970816; doi:10.1371/journal.pmed.1002096)
Supplement: S11 Table — (DOC) [file pmed.1002096.s019.doc]

| **Supplemental Table 11.** Sensitivity Analysis: Ebola period begins on May 29, when Liberia’s second wave began. N=898 | | | | | | | | |
| --- | --- | --- | --- | --- | --- | --- | --- | --- |
|  | **Unadjusted Model** | | **Multivariable Model 1** | | **Multivariable Model 2** | | **Multivariable Model 3** | |
|  | OR (95% CI) | p | AOR (95% CI) | p | AOR (95% CI) | p | AOR (95% CI) | p |
|  |  |  |  |  |  |  |  |  |
| Ebola period | 0.67 (0.49-0.92) | 0.014 | 0.71 (0.51-0.98) | 0.039 | 0.71 (0.51-0.99) | 0.046 | 0.71 (0.51-1.00) | 0.050 |
| Household wealth |  |  | 1.67 (1.29-2.17) | <0.001 | 1.25 (0.98-1.58) | 0.068 | 1.25 (0.99-1.59) | 0.062 |
| Maternal education |  |  |  |  |  |  |  |  |
| None |  |  | Ref. | Ref. | Ref. | Ref. | Ref. | Ref. |
| Primary only |  |  | 1.19 (0.81-1.75) | 0.369 | 1.10 (0.76-1.58) | 0.611 | 1.06 (0.72-1.56) | 0.769 |
| Secondary or higher |  |  | 1.44 (0.80-2.59) | 0.221 | 1.54 (0.84-2.82) | 0.155 | 1.53 (0.81-2.92) | 0.190 |
| Bassa language speaker |  |  |  |  | 0.77 (0.50-1.18) | 0.226 | 0.76 (0.49-1.18) | 0.215 |
| Distance from health facility |  |  |  |  |  |  |  |  |
| Per km, up to 10km |  |  |  |  | 0.85 (0.78-0.92) | <0.001 | 0.85 (0.78-0.92) | <0.001 |
| Per km, 10 to 21km |  |  |  |  | 1.00 (0.93-1.08) | 0.991 | 1.00 (0.93-1.08) | 0.966 |
| Per km, 21km and over |  |  |  |  | 0.91 (0.82-1.00) | 0.054 | 0.91 (0.83-1.01) | 0.072 |
| Maternal age at birth |  |  |  |  |  |  |  |  |
| First quartile |  |  |  |  |  |  | Ref. | Ref. |
| Second quartile |  |  |  |  |  |  | 0.73 (0.46-1.18) | 0.195 |
| Third quartile |  |  |  |  |  |  | 0.72 (0.48-1.08) | 0.107 |
| Fourth quartile |  |  |  |  |  |  | 0.75 (0.48-1.18) | 0.214 |
| Mother is married |  |  |  |  |  |  | 1.04 (0.63-1.70) | 0.887 |
| Birth order |  |  |  |  |  |  |  |  |
| 1^st^ |  |  |  |  |  |  | Ref. | Ref. |
| 2^nd^ or 3^rd^ |  |  |  |  |  |  | 0.88 (0.63-1.27) | 0.492 |
| 4^th^ or higher |  |  |  |  |  |  | 1.15 (0.78-1.69) | 0.488 |
| Rainy season birth |  |  |  |  |  |  | 0.87 (0.63-1.21) | 0.408 |
|  | | | | | | | | |
