# Supplementary material for: Facility-Based Delivery during the Ebola Virus Disease Epidemic in Rural Liberia: Analysis from a Cross-Sectional, Population-Based Household Survey
Source: PLoS Med. 2016 Aug 2;13(8):e1002096. doi: 10.1371/journal.pmed.1002096 (PMC4970816; doi:10.1371/journal.pmed.1002096)
Supplement: S12 Table — (DOC) [file pmed.1002096.s020.doc]

| **Supplemental Table 12.** Sensitivity Analysis: Ebola period begins on July 15, by which point Ebola was a national epidemic. N=898 | | | | | | | | |
| --- | --- | --- | --- | --- | --- | --- | --- | --- |
|  | **Unadjusted Model** | | **Multivariable Model 1** | | **Multivariable Model 2** | | **Multivariable Model 3** | |
|  | OR (95% CI) | p | AOR (95% CI) | p | AOR (95% CI) | p | AOR (95% CI) | p |
|  |  |  |  |  |  |  |  |  |
| Ebola period | 0.68 (0.50-0.92) | 0.015 | 0.73 (0.53-1.02) | 0.066 | 0.71 (0.51-0.98) | 0.041 | 0.69 (0.49-0.97) | 0.031 |
| Household wealth |  |  | 1.68 (1.29-2.18) | <0.001 | 1.24 (0.98-1.58) | 0.071 | 1.25 (0.99-1.59) | 0.064 |
| Maternal education |  |  |  |  |  |  |  |  |
| None |  |  | Ref. | Ref. | Ref. | Ref. | Ref. | Ref. |
| Primary only |  |  | 1.18 (0.80-1.73) | 0.393 | 1.09 (0.76-1.58) | 0.640 | 1.05 (0.72-1.54) | 0.791 |
| Secondary or higher |  |  | 1.44 (0.80-2.59) | 0.222 | 1.54 (0.84-2.82) | 0.162 | 1.53 (0.80-2.92) | 0.196 |
| Bassa language speaker |  |  |  |  | 0.77 (0.50-1.18) | 0.221 | 0.76 (0.49-1.18) | 0.213 |
| Distance from health facility |  |  |  |  |  |  |  |  |
| Per km, up to 10km |  |  |  |  | 0.85 (0.78-0.92) | <0.001 | 0.85 (0.78-0.92) | <0.001 |
| Per km, 10 to 21km |  |  |  |  | 1.00 (0.93-1.08) | 0.988 | 1.00 (0.93-1.08) | 0.962 |
| Per km, 21km and over |  |  |  |  | 0.91 (0.83-1.00) | 0.062 | 0.91 (0.83-1.01) | 0.080 |
| Maternal age at birth |  |  |  |  |  |  |  |  |
| First quartile |  |  |  |  |  |  | Ref. | Ref. |
| Second quartile |  |  |  |  |  |  | 0.73 (0.46-1.17) | 0.189 |
| Third quartile |  |  |  |  |  |  | 0.71 (0.47-1.06) | 0.092 |
| Fourth quartile |  |  |  |  |  |  | 0.74 (0.47-1.17) | 0.200 |
| Mother is married |  |  |  |  |  |  | 1.03 (0.63-1.70) | 0.894 |
| Birth order |  |  |  |  |  |  |  |  |
| 1^st^ |  |  |  |  |  |  | Ref. | Ref. |
| 2^nd^ or 3^rd^ |  |  |  |  |  |  | 0.89 (0.61-1.28) | 0.507 |
| 4^th^ or higher |  |  |  |  |  |  | 1.16 (0.78-1.72) | 0.453 |
| Rainy season birth |  |  |  |  |  |  | 0.85 (0.61-1.17) | 0.301 |
|  | | | | | | | | |
