# Supplementary material for: Facility-Based Delivery during the Ebola Virus Disease Epidemic in Rural Liberia: Analysis from a Cross-Sectional, Population-Based Household Survey
Source: PLoS Med. 2016 Aug 2;13(8):e1002096. doi: 10.1371/journal.pmed.1002096 (PMC4970816; doi:10.1371/journal.pmed.1002096)
Supplement: S14 Table — (DOC) [file pmed.1002096.s022.doc]

| **Supplemental Table 14.** Sensitivity Analysis: Random redistribution of heaped birth dates from first day of month. N=899 | | | | | | | | |
| --- | --- | --- | --- | --- | --- | --- | --- | --- |
|  | **Unadjusted Model** | | **Multivariable Model 1** | | **Multivariable Model 2** | | **Multivariable Model 3** | |
|  | OR (95% CI) | p | AOR (95% CI) | p | AOR (95% CI) | p | AOR (95% CI) | p |
|  |  |  |  |  |  |  |  |  |
| Ebola period | 0.67 (0.49-0.91) | 0.012 | 0.71 (0.51-0.99) | 0.045 | 0.71 (0.50-0.99) | 0.046 | 0.71 (0.50-0.99) | 0.043 |
| Household wealth |  |  | 1.67 (1.29-2.17) | <0.001 | 1.25 (0.98-1.58) | 0.069 | 1.25 (0.99-1.59) | 0.062 |
| Maternal education |  |  |  |  |  |  |  |  |
| None |  |  | Ref. | Ref. | Ref. | Ref. | Ref. | Ref. |
| Primary only |  |  | 1.19 (0.81-1.75) | 0.365 | 1.10 (0.76-1.58) | 0.613 | 1.05 (0.72-1.55) | 0.784 |
| Secondary or higher |  |  | 1.44 (0.80-2.60) | 0.216 | 1.55 (0.84-2.83) | 0.155 | 1.53 (0.80-2.92) | 0.193 |
| Bassa language speaker |  |  |  |  | 0.77 (0.50-1.17) | 0.217 | 0.76 (0.49-1.18) | 0.213 |
| Distance from health facility |  |  |  |  |  |  |  |  |
| Per km, up to 10km |  |  |  |  | 0.85 (0.78-0.92) | <0.001 | 0.85 (0.78-0.92) | <0.001 |
| Per km, 10 to 21km |  |  |  |  | 1.00 (0.93-1.08) | 0.985 | 1.00 (0.93-1.08) | 0.958 |
| Per km, 21km and over |  |  |  |  | 0.91 (0.83-1.00) | 0.059 | 0.91 (0.83-1.01) | 0.072 |
| Maternal age at birth |  |  |  |  |  |  |  |  |
| First quartile |  |  |  |  |  |  | Ref. | Ref. |
| Second quartile |  |  |  |  |  |  | 0.73 (0.46-1.18) | 0.196 |
| Third quartile |  |  |  |  |  |  | 0.71 (0.48-1.07) | 0.101 |
| Fourth quartile |  |  |  |  |  |  | 0.75 (0.47-1.18) | 0.209 |
| Mother is married |  |  |  |  |  |  | 1.04 (0.63-1.70) | 0.880 |
| Birth order |  |  |  |  |  |  |  |  |
| 1^st^ |  |  |  |  |  |  | Ref. | Ref. |
| 2^nd^ or 3^rd^ |  |  |  |  |  |  | 0.88 (0.61-1.28) | 0.507 |
| 4^th^ or higher |  |  |  |  |  |  | 1.16 (0.78-1.71) | 0.461 |
| Rainy season birth |  |  |  |  |  |  | 0.87 (0.63-1.19) | 0.374 |
|  | | | | | | | | |
