# Supplementary material for: Facility-Based Delivery during the Ebola Virus Disease Epidemic in Rural Liberia: Analysis from a Cross-Sectional, Population-Based Household Survey
Source: PLoS Med. 2016 Aug 2;13(8):e1002096. doi: 10.1371/journal.pmed.1002096 (PMC4970816; doi:10.1371/journal.pmed.1002096)
Supplement: S1 Text — (DOCX) [file pmed.1002096.s023.docx]

**Analysis History**

The analytical methods for this analysis are described in the manuscript’s methods section and further described here. As noted in the manuscript, the data for this study were collected as a baseline assessment prior to the implementation of a community health worker program in Rivercess County, Liberia.

The idea for the current study was motivated by our prior work to discern determinants of maternal and child health in rural Liberia. The Ebola virus disease (EVD) epidemic represented a substantial shock to the health system that was predicted to impact maternal health. We designed this analysis in May 2015, towards the end of the epidemic, to help estimate the collateral effects of the epidemic on maternal health in rural Liberia. In particular, we were concerned about its implications for facility-based delivery based on anecdotal reports our health care staff had received about mothers forgoing facility deliveries either due to fears about EVD transmission or because of social interruptions.

Our data consist primarily of a reduced set of standardized DHS survey items. We analyzed facility-based delivery as the outcome of interest because it was the best available maternal or child health indicator for which we could reliably construct a retrospective cohort. The only other potential outcome, childhood vaccination, was complicated by very low levels of vaccine card retention, preventing valid estimates of changes in vaccination rates over time. We determined the data for our primary predictor of interest, calendar period prior to or during the Ebola epidemic, before analyses were conducted through consultation with clinical and programmatic colleagues working in rural Liberia. We chose to include as potential control variables any available variable associated with FBD in our a study of facility delivery we had conducted in rural Liberia (<http://www.ncbi.nlm.nih.gov/pubmed/26207180>) or any variable identified from our review of the literature that was correlated with facility based delivery (referenced in the manuscript). Model construction, statistical analyses, and sensitivity analyses were determined simultaneously, subsequently unaltered, and conducted as we reported them in the manuscript. The analyses reported in supplements 4 and 6 were added at the request of reviewers.
